# Model scenarios for cell cycle re-entry in Alzheimer's disease

**DOI:** 10.1016/j.isci.2022.104543

**Published:** 2022-06-07

**Authors:** Nishtha Pandey, P.K. Vinod

**Affiliations:** 1 Center for Computational Natural Sciences and Bioinformatics, International Institute of Information Technology, Hyderabad 500032 India

**Keywords:** Bioinformatics, Biological sciences, Neuroscience, Systems biology, Systems neuroscience

## Abstract

Alzheimer's disease (AD) is the most prevalent neurodegenerative disease. Aberrant production and aggregation of amyloid beta (Aβ) peptide into plaques is a frequent feature of AD, but therapeutic approaches targeting Aβ accumulation fail to inhibit disease progression. The approved cholinesterase inhibitor drugs are symptomatic treatments. During human brain development, the progenitor cells differentiate into neurons and switch to a postmitotic state. However, cell cycle re-entry often precedes loss of neurons. We developed mathematical models of multiple routes leading to cell cycle re-entry in neurons that incorporate the crosstalk between cell cycle, neuronal, and apoptotic signaling mechanisms. We show that the integration of multiple feedback loops influences disease severity making the switch to pathological state irreversible. We observe that the transcriptional changes associated with this transition are also characteristics of the AD brain. We propose that targeting multiple arms of the feedback loop may bring about disease-modifying effects in AD.

## Highlights

• Developed mathematical models of cell cycle re-entry in Alzheimer's disease (AD)
• Integration of multiple feedback loops drives irreversible transition to AD
• Predicted transcriptional dysregulation is validated using AD gene expression data
• Inhibition of self-amplifying feedback loops brings about disease-modifying effects

## Graphical abstract

**Figure:**
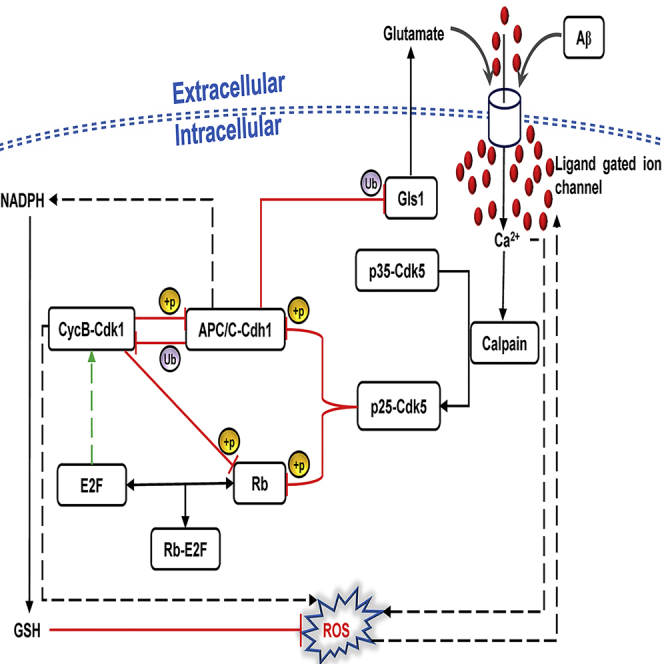

## Introduction

Neurodegeneration refers to the gradual deterioration of neuronal structure and function, leading to loss of cognitive abilities and dementia. According to the recent reports from the World Health Organization (WHO), approximately 55 million people suffer from neurodegenerative disease worldwide, with an annual rise of about 10 million. AD is the most prevalent neurodegenerative disease contributing to 60%–70% of the cases (Dementia, 2021). These cases primarily belong to two subgroups, familial AD (FAD) and sporadic AD (SAD). FAD is usually associated with mutations in the amyloid precursor protein (APP) gene or its processing enzymes (PSEN1 and PSEN2). These mutants exacerbate the accumulation of Aβ peptide and plaque formation in the extracellular region between neurons. Clinical manifestations of neurodegeneration usually appear at an earlier age. However, FAD is a less frequent form affecting only about 5% of the patients (Bali et al., 2012). The etiology of SAD is more complex and attributes to multiple risk factors such as age, brain injury, inefficient removal of Aβ, epsilon4 allele form of apolipoprotein E (APOE), midlife hypertension, high cholesterol, and obesity but none of these serves as a determining factor (Association, 2019; Dorszewska et al., 2016; Piaceri et al., 2013). AD onset is late if the cognitive impairment symptoms appear among the elderly post 65 years (Bali et al., 2012); the frequency increases from 3% among people aged 65–74 years to 32% among 85 years and above. However, the onset of systemic changes like Aβ accumulation precedes the manifestation of dementia by more than a decade (Association, 2019).

AD is a multifactorial disease, and in most cases, is not pinned down to a specific root. Numerous factors have been investigated for their potential as a causative agent and several hypotheses have been proposed to provide the mechanistic detail of AD. Accordingly, various therapeutic approaches targeting the underlying molecular players have been tested (Breijyeh and Karaman, 2020; Liu et al., 2019). One of the earliest theories put forward is the cholinergic deficit hypothesis which attributes the loss of cholinergic neurons and reduction in acetylcholine synthesis to cognitive impairment in AD pathology (Davies and Maloney, 1976). Therefore, cholinesterase inhibitor (ChEI) drugs have been in use for AD for decades now. However, this class of drug is largely successful as a symptomatic therapy and has failed to fetch an overall promising disease-modifying effect in AD pathogenesis (Liu et al., 2019; Siegfried, 1993). ChEI manages AD symptoms by inhibiting cholinesterase, the enzyme that breaks down choline neurotransmitters. Its inability to inhibit disease progression, in general, suggests cholinergic neuronal atrophy is rather a consequence and not a mechanism of neurodegeneration (Haake et al., 2020; Sharma, 2019).

Another theory is the amyloid cascade hypothesis (ACH) that Hardy and Higgins proposed to describe AD pathogenesis in 1992. They hypothesized Aβ aggregates, the main constituent of amyloid plaque, as the causative agent of AD and other abnormalities like hyperphosphorylation of the microtubule-associated protein tau (MAPT/tau), the formation of intracellular neurofibrillary tangles (NFT), cell loss, and dementia follow as subsequent effects of Aβ accumulation (Hardy and Higgins, 1992). This hypothesis is supported by the driver mutations in FAD as well as genome-wide association studies (GWAS) in SAD. The risk genes identified in GWAS include SORL1, CLU, and APOE, which participate in the sorting and trafficking of proteins, preventing aggregate formation and clearance of deposits (Reitz, 2012). Other studies reported Aβ peptides may exist in multiple neurotoxic forms. Hence, since the proposal of ACH, numerous studies have explored the neurodegenerative effects of different forms of aggregated amyloid fibrils and soluble Aβ oligomers (Ferreira et al., 2015; Lambert et al., 1998). Soluble Aβ oligomers are commonly found in AD brains and are more neurotoxic due to their diffusible nature. They can bind a wide array of protein and non-protein neuronal receptors including glutamate receptors and turn on downstream signaling processes. It can eventually lead to hyperphosphorylation of tau, dysregulation of the neuronal processes, synaptic degeneration, and loss of neurons (Ferreira et al., 2015). Inhibitors interfering with APP processing, Aβ aggregation, and therapies facilitating Aβ clearance are most frequently tested in clinical trials. However, despite highly efficient removal of Aβ from plasma and cerebrospinal fluid, they have failed to fetch promising results in clinical trials (Liu et al., 2019). The failure of Aβ plaque clearance therapies points toward the self-sustaining role of downstream effectors that regulate disease progression post Aβ exposure. Additionally, worsening cognitive decline in some case (NCT03131453) may be attributed to the physiological role of Aβ in long-term potentiation at lower concentration (picomolar) (Kent et al., 2020). Hence, the amyloid cascade hypothesis has been reviewed critically in time and again (Rapoport et al., 2002; Ricciarelli and Fedele, 2017; Sengupta et al., 2016). Rather than exploring series of events leading to a cascade, the need to identify downstream self-amplifying mechanisms that regulate AD progression and sustain pathological manifestation in the absence of the initial trigger has been felt (Doig, 2018; García-Ayllón et al., 2011; Rao et al., 2020).

Expression of cell cycle activators is significantly upregulated in postmortem samples from degenerating regions of AD brain (Absalon et al., 2013; Huang et al., 2019; Joseph et al., 2020; Marlier et al., 2020; Nagy et al., 1997; Smith et al., 1999; Van Leeuwen et al., 2015; Vincent et al., 1997). These proteins also show up in individuals with mild cognitive impairment and minor Aβ plaque load (Yang et al., 2003). Similar finding is recapitulated in transgenic AD mice model where appearance of cell cycle events in vulnerable regions of brain precedes pathological markers (Yang et al., 2006). Furthermore, neuronal cell cycle re-entry transgenic mice model manifests NFT and amyloid pathology (Park et al., 2007), whereas double transgenic mice exhibit development of enhanced AD-associated feature like tau pathology and neurodegeneration than transgenic AD mice model (Barrett et al., 2021). In line with the animal models, overexpression of cell cycle activators/oncogenes induces AD-like changes, whereas inhibitors of cyclin-dependent kinase (Cdk) rescue cell division and subsequent apoptosis in neuronal cell lines (Giovanni et al., 1999; McShea et al., 2007; Veas-Pérez De Tudela et al., 2015b). Furthermore, pathological phosphorylation of tau by Cdks increases its stability leading to destabilization of microtubular dynamics, synaptic loss, and neuronal dysfunction (Baumann et al., 1993; Kametani and Hasegawa, 2018; Lee et al., 2009a; Pei et al., 2002). These observations suggest cell cycle re-entry not only precedes neuron loss but it also mediates and escalates the disease progression. It appears counterintuitive since neurons are known to exit proliferation permanently and maintain a postmitotic, differentiated state after human brain development (Allnutt et al., 2020). High levels of Cdk inhibitors (CDKI), retinoblastoma protein (Rb), and anaphase-promoting complex/cyclosome (APC/C)-Cdh1 ensure a non-dividing state (Delgado-Esteban et al., 2013; Frade and Ovejero-Benito, 2015; Harmey et al., 2009; Marlier et al., 2020). On the other hand, cyclins perform alternate functions such as the regulation of synaptic plasticity in neurons (Odajima et al., 2011; Yan and Ziff, 1995). Cdk5 is the most abundant member of the Cdk family in neurons, and it forms complexes with p35 and p39. In contrast to the function of other Cdks, it participates in cell cycle suppression. Cdk5 is also involved in brain development, cortical neuron migration, and microtubule regulation (Allnutt et al., 2020; Cicero and Herrup, 2005; Marlier et al., 2020; Shah and Lahiri, 2014).

In this work, we studied different model scenarios for cell cycle re-entry in neurons. Mathematical models of control circuits leading to cell cycle-regulated neuronal apoptosis (CRNA) were developed. We show that the integration of multiple feedback loops influences the severity of disease and makes the switch to pathological state irreversible. Based on the model predictions, we propose that simultaneous clearance of extracellular Aβ aggregates and inhibition of multiple arms of the feedback loop may bring about disease-modifying effects in moderate and severe AD. The mathematical model presented here is the first such attempt to mechanistically link cell cycle re-entry with neuronal apoptosis.

### Molecular network reconstruction of CRNA

We reconstructed CRNA control circuits based on the available information in the literature. The critical cell cycle regulators involved in the control of CRNA include APC/C-Cdh1, Rb, E2F, p35/p25-Cdk5, Cyclin-Cdk, and CDKI (p21 and p27). These proteins emerged as important players since they control a multitude of substrates. APC/C-Cdh1 and Rb maintain the neurons in a nondividing, differentiated state, whereas Cyclin-Cdk and E2F drive cell cycle re-entry. Cdk5 and p27, on the other hand, act as a double-edged sword. Cdk5 activity is regulated in neurons by mechanisms involving autophosphorylation and rapid degradation (Shah and Lahiri, 2014). Cdk5-deficient mice (Cdk5^−/−^) fail to develop normally and die perinatally with multiple abnormalities in the cerebral cortex, hippocampus, and cerebellum. In contrast, high Cdk5 activity contributes to the complex etiology of AD by hyperphosphorylation of various physiological and non-physiological substrates (Allnutt et al., 2020). Under physiological conditions, p27, a member of the Kip family of CDKI, also contributes to sustaining mature neurons in the differentiated state in a manner analogous to APC/C-Cdh1, Rb, and Cdk5. Gene silencing experiments targeting p27 promote cell cycle re-entry (Rb phosphorylation) and apoptosis in rat cortical neurons. Inhibitors of Cdk rescue this effect of p27 silencing (Akashiba et al., 2006). However, immunohistology data from AD brain report accumulation of p27 in the cytosol of both NFT bearing and histologically indistinguishable neurons (Ogawa et al., 2003). A buildup of cytosolic p27 in AD seems to contradict with the canonical, neuroprotective role of p27 in differentiated neurons. We describe three network modules involving these components in the control of CRNA.

### Module 1: Aβ-induced hyperactivation of extracellular signal-regulated kinases (ERK) in neurons

**Figure 1:**
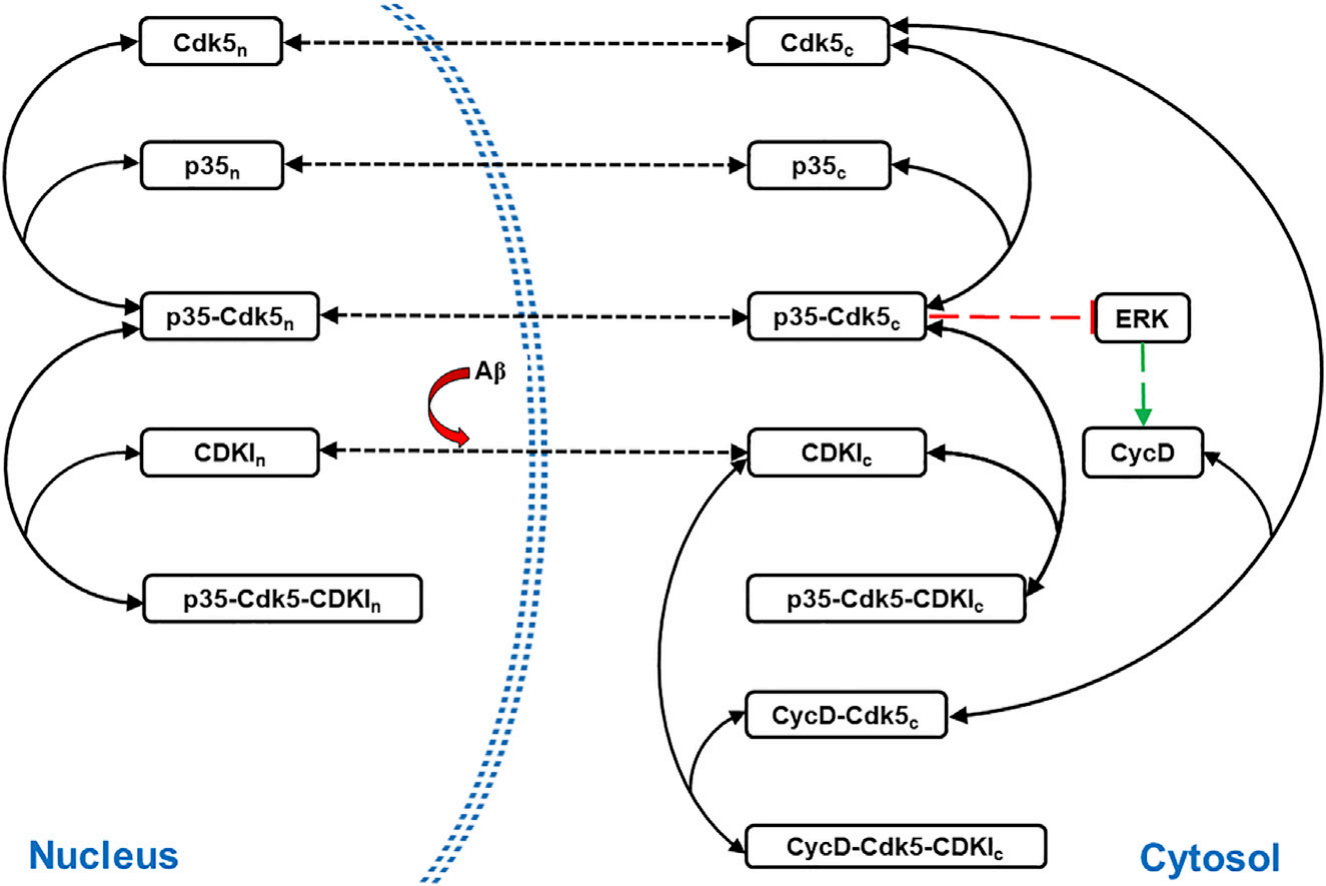
Wiring diagram depicting competition between p35 and CycD which controls ERK activity on Aβ exposure Aβ controls the nuclear export of p27 (CDKI). The nucleocytoplasmic translocation is shown by discontinuous double-headed arrow. The red color line with blunt end indicates inhibition and green arrow indicates transcriptional activation, discontinuous lines indicate indirect control. Solid lines with double-headed arrows denote reversible association and dissociation of complexes.

The temporal profile of ERK activity determines cell fate; a sustained but low activity promotes differentiated state of neurons (Chambard et al., 2007; Chen et al., 2012; Ryu et al., 2016). In differentiated neurons, the p35-Cdk5 complex indirectly limits the sustained ERK activity (Figure 1) by inhibiting its upstream regulator MAP kinase kinase-1 (MEK-1) through phosphorylation (Sharma et al., 2002). Cyclin D (CycD) competes with p35 for Cdk5 binding in the presence of Aβ and thereby intervenes with the physiological, neuroprotective function of Cdk5. Loss of p35-Cdk5 activity dysregulates the MEK-ERK signaling pathway by relieving its repression. Hyperactivated ERK increases CycD expression further (Chambard et al., 2007; Modi et al., 2012, 2015). However, CycD, p35, and Cdk5 are abundant in postmitotic neurons (Allnutt et al., 2020; Yan and Ziff, 1995). This raises the question of how the binding partner of Cdk5 switches from p35 to CycD on Aβ exposure. Cdk5 carries nuclear export signal and intrinsically tends to be localized outside nucleus (J. Zhang et al., 2010a). In the resting neurons, p27 (CDKI) compartmentalizes p35-Cdk5 to the nucleus by trimer complex formation. Aβ exposure exports p27 to the cytoplasm (J. Zhang et al., 2010a), and the relative compartment-wise distribution of these proteins changes. CycD primarily localizes in the cytoplasm of differentiated neurons (Sumrejkanchanakij et al., 2003). Cytosolic p27 stabilizes its association with Cdk5. siRNA targeted against p27 rescues p35-Cdk5 association and is neuroprotective (Jaiswal and Sharma, 2017). Hence, Aβ, through nuclear export of p27, topples the p35-Cdk5 balance to CycD-Cdk5 state leading to ERK hyperactivation and CycD accumulation.

### Module 2: Intracellular Ca^2+^-dependent APC/C-Cdh1 inactivation, Rb hyperphosphorylation, and E2F induction in neurons

**Figure 2:**
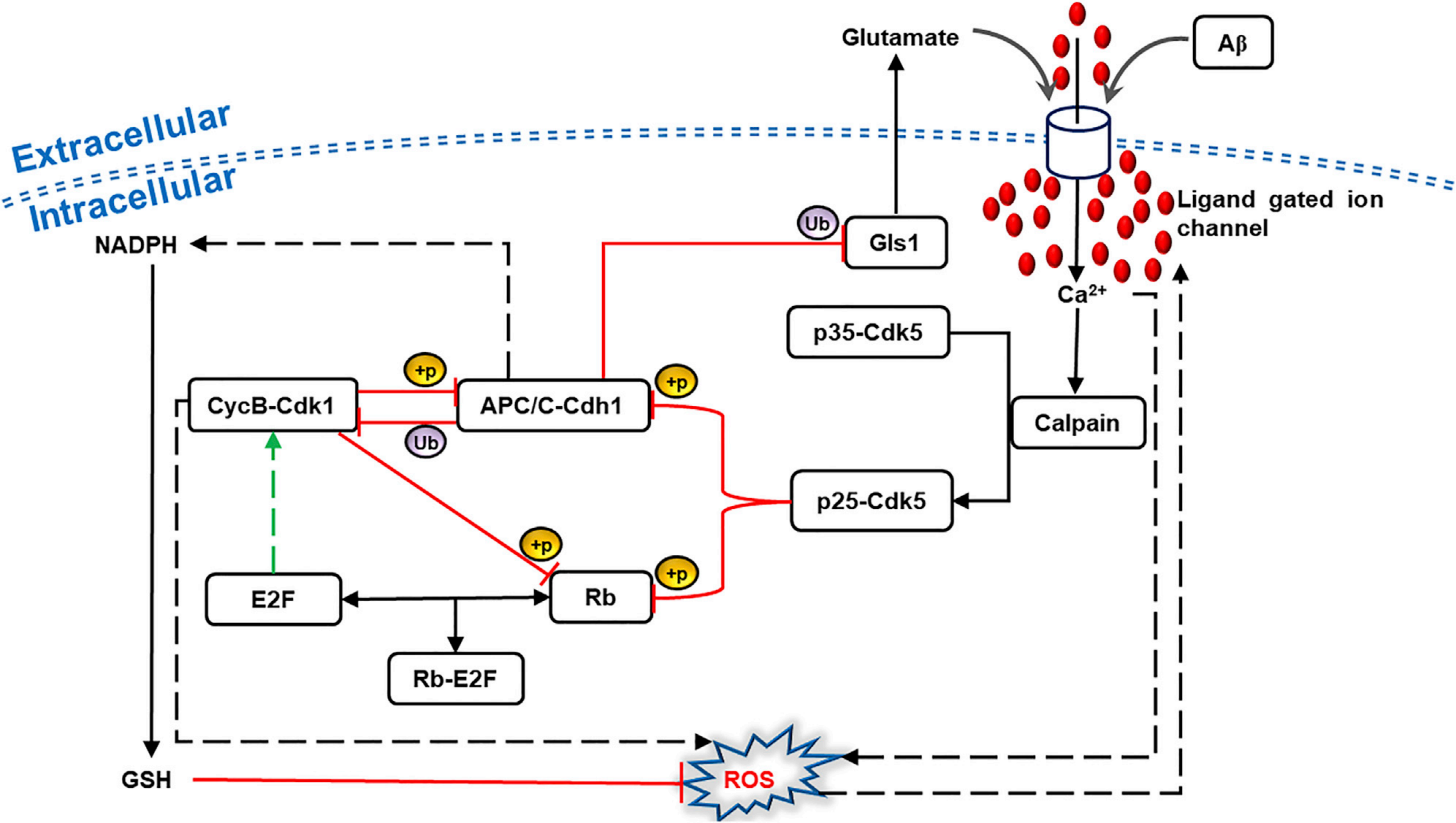
Wiring diagram depicting APC/C-Cdh1’s role in neuroprotection The red color line with blunt end indicates inhibition. Arrows represent activation with green color indicating transcriptional regulation. The discontinuous lines indicate indirect control. Solid lines with double-headed arrows denote reversible association and dissociation of complexes. The underlying mechanism for inhibition is shown with +p for phosphorylation and Ub for ubiquitination.

Rb and APC/C-Cdh1 maintain a non-proliferating, differentiated state of neurons. Rb suppresses the cell cycle by stoichiometric inhibition of the E2F transcription factor. APC/C-Cdh1, on the other hand, belongs to the E3 ubiquitin ligase family that gets activated at the end of mitosis in cycling cells and remains active till the G1/S transition of the next cycle (Cappell et al., 2016). In the quiescent (G0) and differentiated state, APC/C-Cdh1 suppresses the cell cycle by promoting proteasomal degradation of cell cycle activators (Harper et al., 2002). Glutamate excitotoxicity or Aβ exposure perturbs intracellular Ca^2+^ balance through stimulation of the ligand-gated ion channel present on the membrane of differentiated neurons. Ca^2+^ dysregulation activates calpain-catalyzed cleavage of p35 into p25; p25 has a slower turnover rate which increases kinase activity of Cdk5 (Ferreira et al., 2015; Lee et al., 2000; Mucke and Selkoe, 2012; Patrick et al., 1999; Veas-Pérez De Tudela et al., 2015b) (Figure 2). p25-Cdk5 inactivates Rb and APC/C-Cdh1 by phosphorylation (Fuchsberger et al., 2016; Futatsugi et al., 2012). While Rb phosphorylation frees E2F and drives the synthesis of cyclins, APC/C-Cdh1 inhibition brings down their degradation. Besides its direct role in cell cycle regulation, APC/C-Cdh1 also regulates the metabolic and redox state of cells. It diverts glycolytic flux toward the pentose phosphate pathway (PPP) through degradation of 6-phosphofructo-2-kinase/fructose-2, 6-bisphosphatase-3 (Pfkfb3). The nicotinamide adenine dinucleotide phosphate (NADPH) molecules produced as a by-product of PPP play a role in generating reduced glutathione (GSH) (Figure 2). GSH maintains redox homeostasis by scavenging reactive oxygen species (ROS) (Herrero-Mendez et al., 2009). The APC/C-Cdh1 function is of prime importance in neurons since the high metabolic rate of the brain makes it susceptible to ROS generation and oxidative stress (Cobley et al., 2018). Furthermore, E2F also contributes to ROS generation by increasing Cyclin B (CycB) accumulation (via. FOXM1) (Fischer et al., 2016; Liao et al., 2018) and APC/C-Cdh1 inactivation (Cappell et al., 2016). Mitochondrially localized CycB-Cdk1 phosphorylates Bcl-xL and interrupts ATP-synthase activity. This leads to enhanced electron leak through the electron transport chain (ETC) and ROS accumulation (Figure 2) (Veas-Pérez De Tudela et al., 2015a). Excessive ROS adversely affects neuronal viability through oxidative DNA damage and apoptosome activation (Redza-Dutordoir and Averill-Bates, 2016). APC/C-Cdh1 additionally manages neuronal activity via modulation of ligand-gated ion channels. Glutaminase (Gls1), an enzyme that catalyzes the conversion of glutamine to glutamate via glutaminolysis pathway, is an APC/C-Cdh1 substrate. In the absence of APC/C-Cdh1, Gls1 activity increases, leading to an increase in glutamate levels. The glutamate excitotoxicity triggers extended periods of receptor stimulation in neurons and dysregulation of intracellular Ca^2+^ (Fuchsberger et al., 2016; Veas-Pérez De Tudela et al., 2015b). Ca^2+^ imbalance causes mitochondrial dysfunction and ROS generation. ROS accumulation alters membrane permeability by lipid peroxidation, and intracellular Ca^2+^ increases further (De Caluwé and Dupont, 2013; Müller et al., 2018; Redza-Dutordoir and Averill-Bates, 2016). Intracellular Ca^2+^ and ROS amplify each other; APC/C-Cdh1 contributes to the amplification through regulation of its substrates described above (Figure 2).

### Module 3: DNA damage-dependent increase in CycD-Cdk4/6 and E2F accumulation in neurons

**Figure 3:**
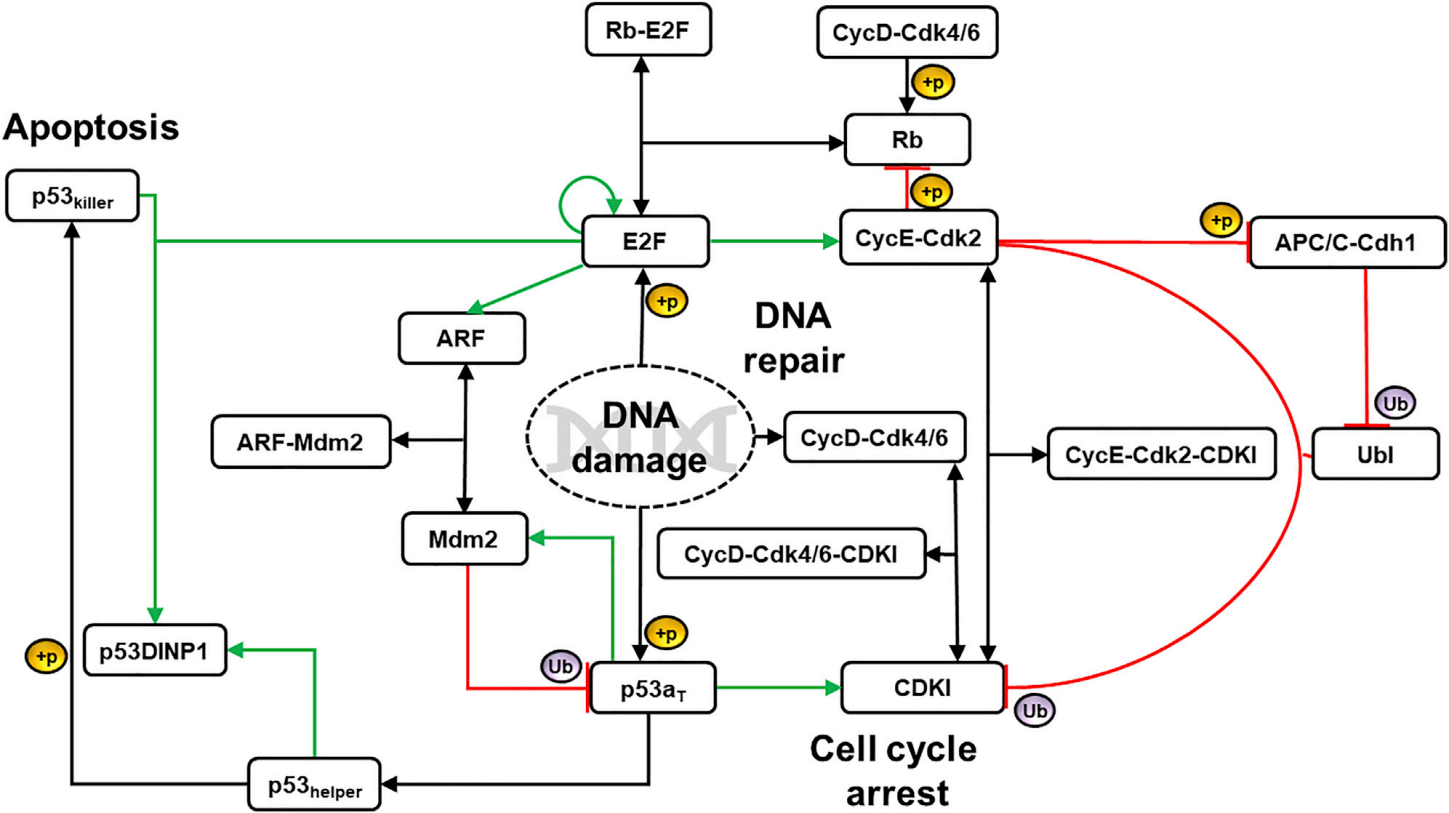
Wiring diagram depicting DNA damage-induced route to cell cycle re-entry and subsequent apoptosis The red color line with blunt end indicates inhibition. Arrows represent activation with green color indicating transcriptional regulation. Double-headed arrows denote reversible association and dissociation of complexes. The underlying mechanism for activation or inhibition is shown with +p for phosphorylation and Ub for ubiquitination.

While the excessive accumulation of ROS itself has the potential to induce apoptosis, oxidative DNA damage magnifies the quantum of neuronal loss (Folch et al., 2011). Furthermore, the source of DNA damage is not limited to oxidative stress; for instance, aberrant p25-Cdk5 activity also amplifies the extent of damage (Mungenast and Tsai, 2011). The stressors may thus be functional alone or in concert. DNA damage induces a series of damage responsive and checkpoint kinases. The damage sensing signals activate CycD-Cdk4/6, stabilize E2F by posttranslational modifications (PTM), and induce its transcription (Castillo et al., 2015; Schwartz et al., 2007; Stevens et al., 2003; Stevens and La Thangue, 2004) (Figure 3). Intracellular Aβ is cytotoxic to neurons through p53-dependent apoptosis pathway (Zhang et al., 2002). It is also known that phosphorylation of p53 by DNA damage sensing kinases leads to the accumulation of active p53 (p53a_T_), which stays predominantly in the p53 helper state (Patil et al., 2013; Tian et al., 2009). In this state, it activates transcription of p21(CDKI) that can block cell cycle re-entry by forming complex with CycD and Cyclin E(CycE). However, neurons may overcome the CDKI barrier and re-enter the cell cycle with an increase in CycD-Cdk4/6 and E2F activity that promote DNA repair and have a protective function. CycD-Cdk4/6 monophosphorylates Rb which then gets hyperphosphorylated by E2F-induced CycE (Narasimha et al., 2014). Rb hyperphosphorylation further relieves stoichiometric inhibition of E2F. Free E2F also promotes APC/C-Cdh1 inactivation, and accumulation of SCF^Skp2-Cks1^(Ubl) complex that promotes CDKI degradation in CycE-Cdk2-dependent manner (Barr et al., 2016; Bashir et al., 2004; Wei et al., 2004). When the damage accumulates beyond repair potential, p53 and E2F coordinate the apoptotic signaling through induction of p53DINP1 that controls the conversion from p53 helper state to p53 killer state. p53 in the killer state activates multiple pro-apoptotic genes (Fischer, 2017; Fridman and Lowe, 2003; Zhang et al., 2009; X. P. Zhang et al., 2010). Thus, neurons may respond to a rise in E2F levels in a graded manner. At a moderate level, p53DINP1 remains low and E2F helps in DNA repair, while at a higher level, it switches on the killer (Zhang et al., 2020). E2F also indirectly stabilizes p53 via modulation of its Mdm2-dependent degradation. It induces tumor suppressor protein ARF that associates with p53 inhibitor Mdm2 and brings down p53 degradation. (Croxton et al., 2002) (Figure 3).

## Results

### Competition between CycD and p35 controls Cdk5 activity

**Figure 4:**
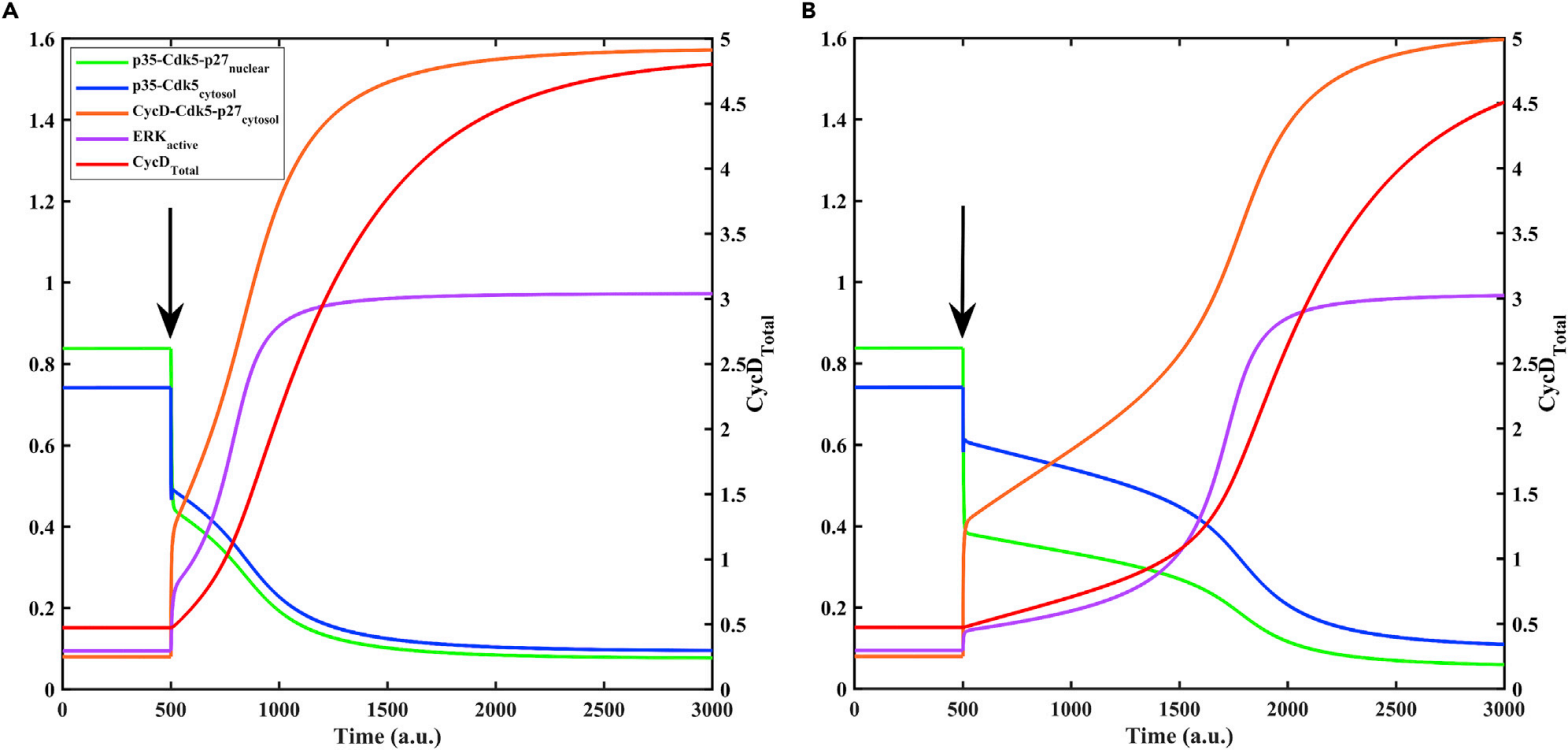
Aβ-mediated activation of MEK-ERK signaling pathway (A) Temporal dynamics of state variables is shown in the presence of Aβ, which induces p27 nuclear export. At t = 500, the Aβ level (marked by arrow) was set to 0.05 from zero. (B) Temporal dynamics of state variables on including the additional effect of Aβ on p35-Cdk5-p27 trimer dissociation (k_dis35cki_ = 10). Relative level of state variable CycD_Total_ is represented on the right y axis. a.u. represents arbitrary units.

We attempted to integrate different experimental findings (Table S1) and present a consensus model for ERK dysregulation in neurons. At first, we captured the initial condition mimicking the differentiated neuron’s resting state. Initially, p35-Cdk5 is almost equally distributed between different compartments (Figure 4A) and the nuclear form stays in p27-bound trimer complex state. This is consistent with the observation of Zhang et al. (2010) (Zhang et al., 2010a), showing that p35-Cdk5 remains evenly distributed in the differentiated neurons as a nucleocytoplasmic protein with its nuclear localization dependent on p27. Conversely, CycD-Cdk5 activity stays limited to cytosol since CycD is largely cytoplasmic in postmitotic neurons (Sumrejkanchanakij et al., 2003).

Aβ exposure (Figure 4A) exports p27 to cytosol, consequently p35 and Cdk5 also move out of nucleus. In the presence of cytosolic p27, CycD competes with p35 for complex formation with Cdk5, resulting in a decrease in p35-Cdk5 activity and an increase in CycD-Cdk5-p27 complex formation (Figure 4A). A reduction in p35-Cdk5 activity leads to hyperactivation of ERK (Figure 4A). An increase in ERK activity leads to the accumulation of CycD that competes with p35 to decrease the p35-Cdk5 activity further. ERK hyperactivity and high CycD (Figure 4A) serve as markers for pathological state and cell cycle re-entry in differentiated neurons. These simulations are consistent with the experimental observations listed in Table S1 (Jaiswal and Sharma, 2017; Modi et al., 2012; J. Zhang et al., 2010a).

**Figure 5:**
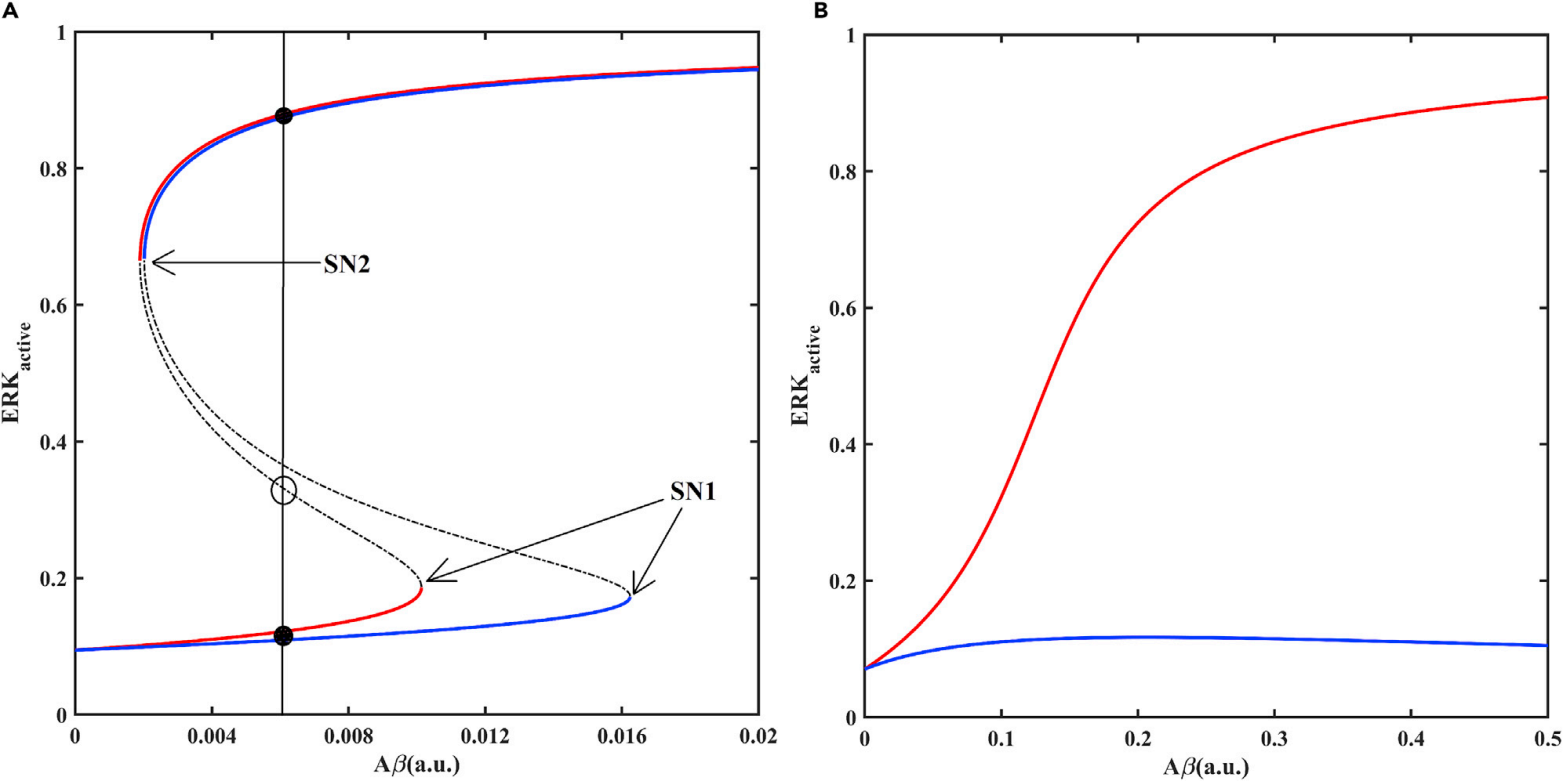
Bifurcation diagram showing the effect of Aβ level on the ERK activation The response curve is shown in the absence (red) and presence (blue) of additional effect of Aβ on p35-Cdk5-p27 trimer dissociation (k_dis35cki_ = 10) in the (A) control and (B) CycD KO (k_scycd_ = 0) conditions. The response curve of ERK shows bistable characteristics (two stable steady states and one unstable steady state for same value of Aβ marked by black filled circles and open circle, respectively). Solid lines represent stable steady states, while dashed line represents unstable steady state. SN1 and SN2 represent the saddle nodes corresponding to ERK activation and inactivation, respectively. a.u. represents arbitrary units.

Furthermore, we show that this positive feedback between CycD and ERK sustains ERK_active_ at pathological levels and gives rise to bistability (Figure 5A). The saddle node 1 (SN1) corresponds to the Aβ threshold for transition from normal to disease state, while SN2 corresponds to the threshold for the transition back to the normal state. Thus, decreasing Aβ does not lead to immediate reversal to the normal state unless its level falls below SN2. MEK inhibition limits CycD to value inadequate for competition with p35 hence the system remains in low ERK activity state and bistability is lost (achieved by k_aerk_ = 0, see Methods S1). Modi et al. (2012) have shown similar cell cycle re-entry rescue experiments in primary cortical cell lines from rat (Table S1) (Modi et al., 2012). Hence, we propose an Aβ-induced cell cycle re-entry mechanism as an ERK bistable switch. Aβ brings about competition between CycD and p35 for Cdk5 association; this turns the switch from low ERK_active_ state to high ERK_active_ state.

The model was also used to simulate other rescue experiments summarized in Table S1. In the absence of p27, CycD fails to sequester Cdk5 away from p35, and ERK activity remains low (Jaiswal and Sharma, 2017). ERK and CycD form two arms of a feedback loop, and transfection of cortical neurons with CycD siRNA or Cdk4/6 inhibitor rescues the effect of Aβ (Giovanni et al., 1999; Modi et al., 2012). However, our model failed to capture the CycD KO phenotype through Aβ effect on cellular localization of p27 only. In the absence of CycD, the competition for complex formation ends and p35-Cdk5-p27 trimer formation occurs in the cytosol, leading to a decrease in p35-Cdk5 activity and an increase in ERK activity (Figure 5B). Thus, we hypothesized that Aβ also directly destabilizes p35-Cdk5-p27 (k_dis35cki_, see Methods S1) by some unknown mechanism. This prevents p35-Cdk5-p27 complex formation and blocks the transition to high ERK_active_ state in the absence of CycD (Figure 5B). Evoking Aβ-dependent trimer dissociation increases nuclear export and cytosolic activity of p35-Cdk5 complex. As a result, ERK suppression strengthens, and the Aβ threshold for ERK_active_ switch shifts to the right (Figure 5A). The dynamics also shows a delay in p35-Cdk5 inactivation and ERK hyperactivation (Figure 4A vs. Figure 4B), which reproduces the temporary neuroprotection provided by Aβ-triggered rise in p35-Cdk5 activity in the cytoplasm (J. Zhang et al., 2010a).

**Figure 6:**
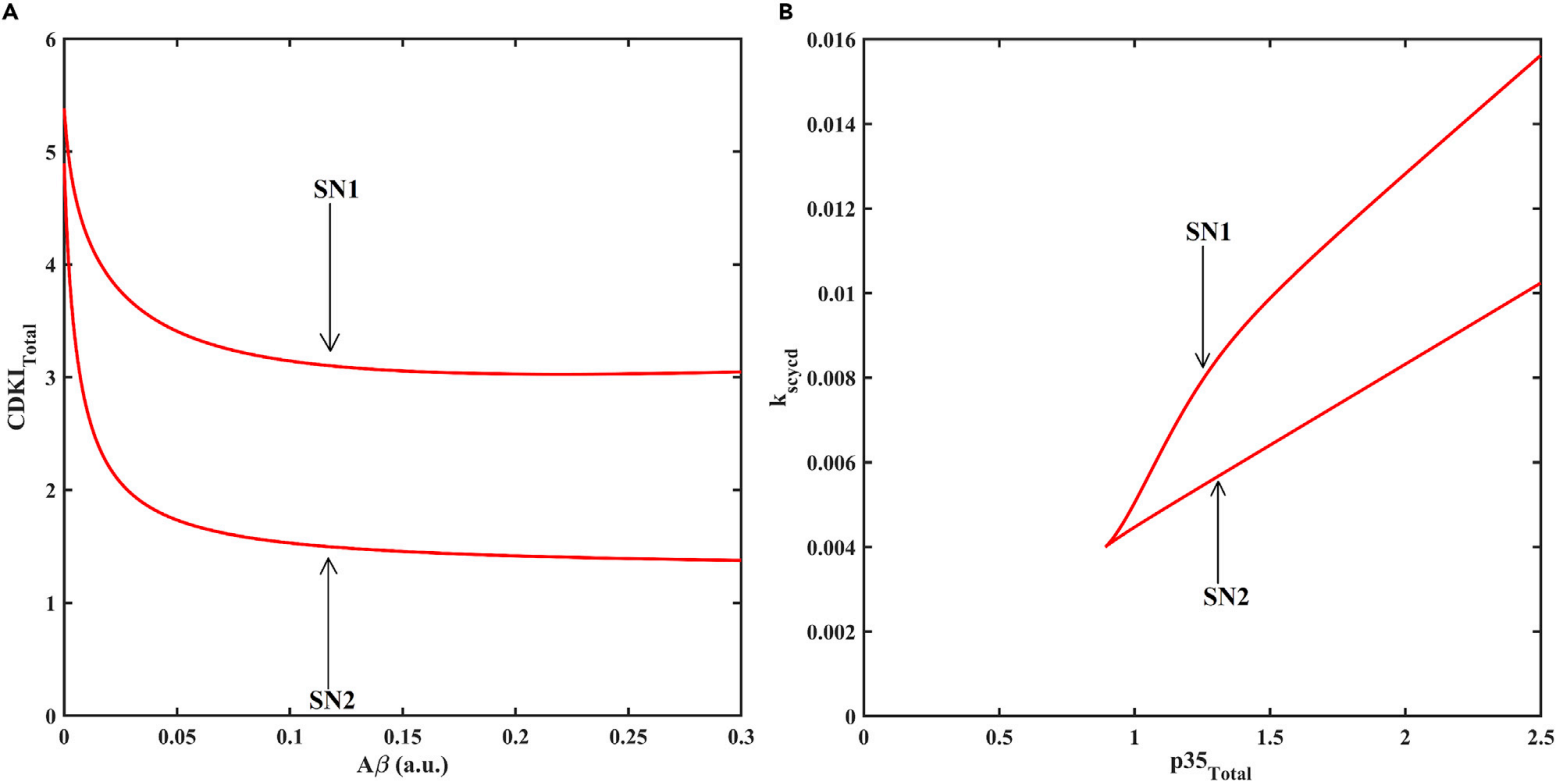
Two-parameter bifurcation plots showing how saddle nodes (SN1 and SN2) of bistable switch corresponding to ERK activation and inactivation, respectively, shift with parametric variation (A) The Aβ threshold for ERK activation and inactivation reduces with increase in p27(CDKI_Total_). (B) An increase in the p35_Total_ increases the CycD requirement (k_scycd_) to activate ERK (Aβ = 0.005). p35_Total_ below a certain threshold fails to suppress ERK and saddle nodes disappear via cusp bifurcation. a.u. represents arbitrary units.

We further studied how the levels of important regulators viz, p27, p35, and CycD affect the regulation of ERK by performing two-parameter bifurcation analysis. We analyzed shift of the two saddle nodes, SN1 and SN2, with respect to second parametric changes. An increase in p27 (CDKI_Total_) levels reduces the Aβ threshold to activate ERK, showing the inverse relationship between Aβ and p27 (Figure 6A). Elevated p27 perturbs the cytosol and nuclear distribution of p35-Cdk5, leading to the sequestration of more p35-Cdk5 in the nucleus and activation of ERK. Therefore, the saddle node shifts to left along the x axis (Aβ). However, nuclear p35-Cdk5-p27 complex formation may still suppress the cell cycle re-entry (J. Zhang et al., 2010b). A decrease in p27 levels increases the Aβ threshold due to an increase in the cytosolic concentration of p35-Cdk5 and stronger inhibition of ERK (Figure 6A). Hence, p27 can perform both anti- and pro-apoptotic functions (Akashiba et al., 2006; Jaiswal and Sharma, 2017) by controlling the p35-Cdk5 nuclear and cytosolic concentration, respectively. Aβ helps in the transition from an anti- to the pro-apoptotic function of p27 by altering the nuclear-cytoplasmic ratio of p35-Cdk5 in the disease state.

We also simulated the relation between p35 and CycD by performing the two-parameter bifurcation analysis for Aβ = 0.005. Figure 6B shows that as the p35 total (p35_Total_) pool increases, the CycD level (k_scycd_, see Methods S1) required for ERK activation also increases. Likewise, for a lower level of p35, the CycD requirement also reduces reflecting the competition (Jaiswal and Sharma, 2017). However, we observed if p35 levels drop beyond a threshold, the p35-Cdk5 activity becomes inadequate to suppress ERK activity. Under such circumstances, ERK remains constitutively hyperactive; the bistable state disappears via cusp bifurcation. Thus, the bistable activation of ERK depends on the competition between CycD and p35 for Cdk5. Decreasing this competition by p35 overexpression bestows neuroprotection.

### Ca^2+^ and ROS nexus in Rb and APC/C-Cdh1 inactivation

In the second module, we studied how different perturbations that converge on Ca^2+^ dysregulation and APC/C-Cdh1 inactivation drive transition to the disease state. This includes exposure to Aβ oligomer, glutamate excitotoxicity, and CycB overexpression (Table S2).

**Figure 7:**
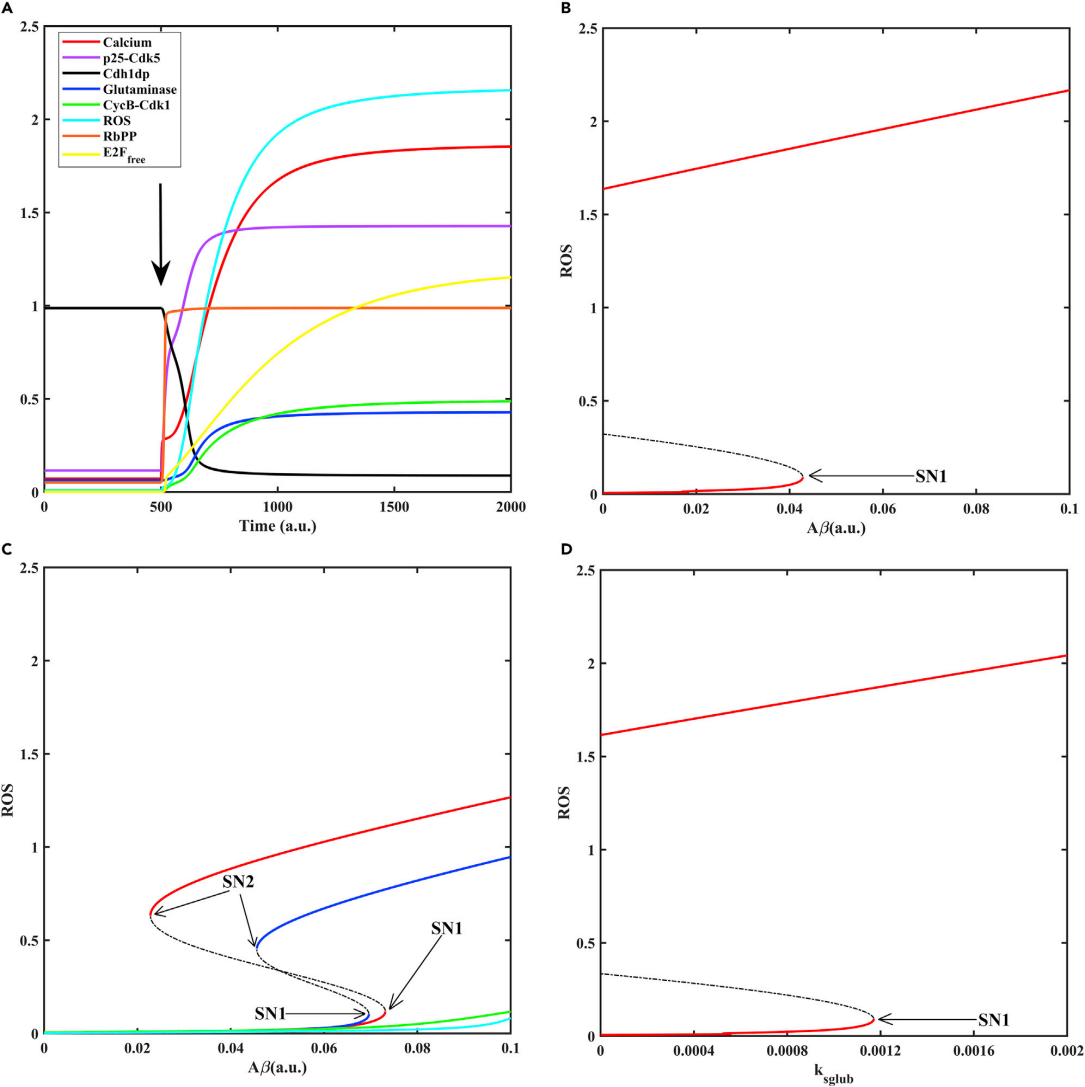
An alternate route to cell cycle re-entry via Rb hyperphosphorylation and APC/C-Cdh1 inactivation (A) Temporal dynamics of state variables is shown in the presence of Aβ by setting its level (marked by arrow) to 0.1 from zero at t = 500. p25-Cdk5 hyperactivity relieves both Rb and APC/C-Cdh1 barrier (RbPP represents inactive, hyperphosphorylated Rb, Cdh1dp represents dephosphorylated active Cdh1). (B) The bifurcation diagram showing the effect of Aβ on ROS accumulation. An irreversible transition to pathological state (oxidative stress) occurs with increase in Aβ level. (C) Blocking the individual feedback loops (red: Gls KO, k_sgls_ = 0; blue: CycB KO, k_scycb_ = 0 = k_scyc_) turned on by APC/C-Cdh1 inactivation leads to a drop in the magnitude of oxidative stress and makes the transitions to pathological state reversible. Inhibition of p25-Cdk5 activity (green: k_sp25_ = 0) or termination of the downstream feedbacks (cyan: k_sgls_ = 0 AND k_scycb_ = 0 = k_scyc_) rescue the transition to pathological state. (D) An increase in glutamate level by increasing k_sglub_ (glutamate excitotoxicity) also shows the irreversible transition to pathological state (oxidative stress). Solid lines represent stable steady states, while dashed line represents unstable steady state. SN1 and SN2 represent the saddle nodes corresponding to APC/C-Cdh1 inactivation and activation, respectively. a.u. represents arbitrary units.

The model mimics the differentiated neuron condition by maintaining Rb, APC/C-Cdh1 active (Figure 7A), Ca^2+^, ROS, E2F targets, and APC/C-Cdh1 substrates low. An increase in Aβ level (Figure 7A) leads to a rise in the influx of Ca^2+^ and activation of p25-Cdk5, which helps to overcome the Rb and APC/C-Cdh1 barrier by phosphorylation (Figure 7A). This leads to amplification in Ca^2+^ and ROS levels by feedback loops (Figure 2). In neurons, a rise in ROS generation has been linked to an increase in the percentage of cells undergoing apoptosis and is rescued by the addition of membrane-permeable antioxidants (Herrero-Mendez et al., 2009; Zhou et al., 1996). Hence, we considered high ROS levels as a marker for pathological state. Rb hyperphosphorylation and APC/C-Cdh1 inactivation mark cell cycle re-entry. We did not observe segregation of two events viz, first hyperphosphorylation of Rb at the restriction point (RP) and later APC/C-Cdh1 inactivation at the G1/S transition as seen with the canonical model of quiescence to proliferation transition with mitogen stimulation (Bashir et al., 2004; Cappell et al., 2016; Narasimha et al., 2014). The temporal separation of RP and G1/S is regulated by the rate at which Cdk2 activity builds up (Cappell et al., 2016; Pandey and Vinod, 2018). The order of G1 phase events is shown to be reversed in the mammary epithelial cell line with a change in the Cdk2 threshold for Rb hyperphosphorylation and APC/C-Cdh1 inactivation in the absence of CycD (Liu et al., 2020). Thus, we speculate our model observation may represent a non-canonical route to cell cycle re-entry in neurodegeneration with no temporal segregation of RP and G1/S transition. p25-Cdk5 is known to inactivate APC/C-Cdh1 without requiring Cdk2 (Veas-Pérez De Tudela et al., 2015b) and hyperphosphorylate Rb with an efficiency comparable to Cdk2 in neurons (Futatsugi et al., 2012).

The bifurcation analysis shows that the system is bistable and undergoes an irreversible transition to pathological state (high ROS) once Aβ levels cross a threshold value (Figure 7B). Such a transition also occurs with CycB overexpression (Figure S1) or glutamate excitotoxicity (Figure 7D). These model features align with the experimental observations listed in Table S2 (Fuchsberger et al., 2016; Veas-Pérez De Tudela et al., 2015a, 2015b). The pathological state arises from Rb and APC/C-Cdh1 inactivation which increase E2F targets and APC/C-Cdh1 substrates. This module includes multiple feedback loops that can switch the system irreversibly into a state of oxidative stress implying the transition becomes independent of Aβ stimulus (Figure 7B). We perturbed the network to study the contribution of individual feedback loops. This includes the mutual antagonism between CycB-Cdk1 and APC/C-Cdh1 (CycB-Cdk1 -----| APC/C-Cdh1 -----| CycB-Cdk1) and APC/C-Cdh1 and Gls1 (APC/C-Cdh1 ---| Gls1 → glutamate → Ca^2+^ → p25-Cdk5 -----| APC/C-Cdh1). Inhibition of Gls1 (k_sgls_ = 0) or CycB-Cdk1 (k_scycb_ = 0 = k_scyc_) (see Methods S2) shifts the saddle nodes to the right and leads to a drop in the upper steady-state values of ROS suggesting that each feedback contributes to the strength of amplification and targeting the feedbacks can delay the onset and disease progression (Figure 7C). The reversible characteristic of pathological state suggests that in the presence of inhibitor of feedback loops, targeted removal of Aβ peptides may alleviate the severity of the disease. The vulnerability of neurons can thus be reduced by glutaminase inhibition, CycB KO, or by the addition of membrane permeable antioxidants (achieved by increasing k_anadphb_), which enhance the ROS scavenging capacity. Our model is consistent with rescue mechanisms that compensate for perturbations such as: addition of APC/C-Cdh1 inhibitor (achieved by making k_acdh1_ = 0), Aβ oligomers, glutamate, and CycB overexpression (Table S2).

We then tested which nodes could be the most potential targets for therapeutic intervention. On evaluating the condition of p25-Cdk5 inhibition (Figure 7C) or collective downstream feedback loop blockade by glutaminase and CycB-Cdk1 inhibition (Figure 7C), we observe the jump to pathological state is lost. Intracellular Ca^2+^ influx initiates APC/C-Cdh1 inactivation by p25-Cdk5 and accumulation of its targets. APC/C-Cdh1 is the central regulator of this network module, and therefore, perturbations around it have a significant effect on the phenotype (Ekshyyan and Aw, 2005) than the direct role of Aβ on Ca^2+^ influx and oxidative stress. This property of the model is in line with the “two-hit hypothesis” proposed for AD. Dual insult in the form of mitogenic stimulation (Rb and APC/C-Cdh1 inactivation) and oxidative stress (depletion of antioxidants and ROS generation) plays a crucial role in disease progression. On a single insult, cells adapt to a new steady but vulnerable state (Zhu et al., 2007). As glutamate excitotoxicity and CycB dysregulation converge on APC/C-Cdh1 deactivation, they may induce the irreversible transition to a pathological state.

### DNA damage-induced cell cycle re-entry: Repair versus apoptosis

Several physiological processes like ATP intensive neuronal activity make normal brain vulnerable to oxidative stress and DNA damage (Castillo et al., 2015; Cobley et al., 2018). Damage sensing kinases elevate p53 and E2F levels in an attempt to arrest and repair (Stevens et al., 2003). However, E2F and p53 can cooperate to trigger apoptosis when the damage is beyond repair (Croxton et al., 2002; Fridman and Lowe, 2003; Stevens and La Thangue, 2004; Wu and Levine, 1994). An unscheduled S-phase entry creates replication stress that escalates the degree of DNA damage (Walton et al., 2019).

In the third module, we explored how the DNA damage-induced cell cycle re-entry occurs in neurons in an attempt to repair, but as a consequence, may lead to apoptosis. The differentiated neuron state is represented by dephosphorylated Rb (RbPP = 0), E2F under stoichiometric inhibition of Rb (E2F_free_ = 0), and dephosphorylated/inactive p53. Accordingly, the repair phase is represented by active p53 helper state, high p21 (CDKI_Total_), and cell cycle re-entry (marked by hyperphosphorylated Rb), whereas apoptotic state is represented by p53 killer state, intermediate p21 (CDKI_Total_), and cell cycle re-entry (marked by hyperphosphorylated Rb).

**Figure 8:**
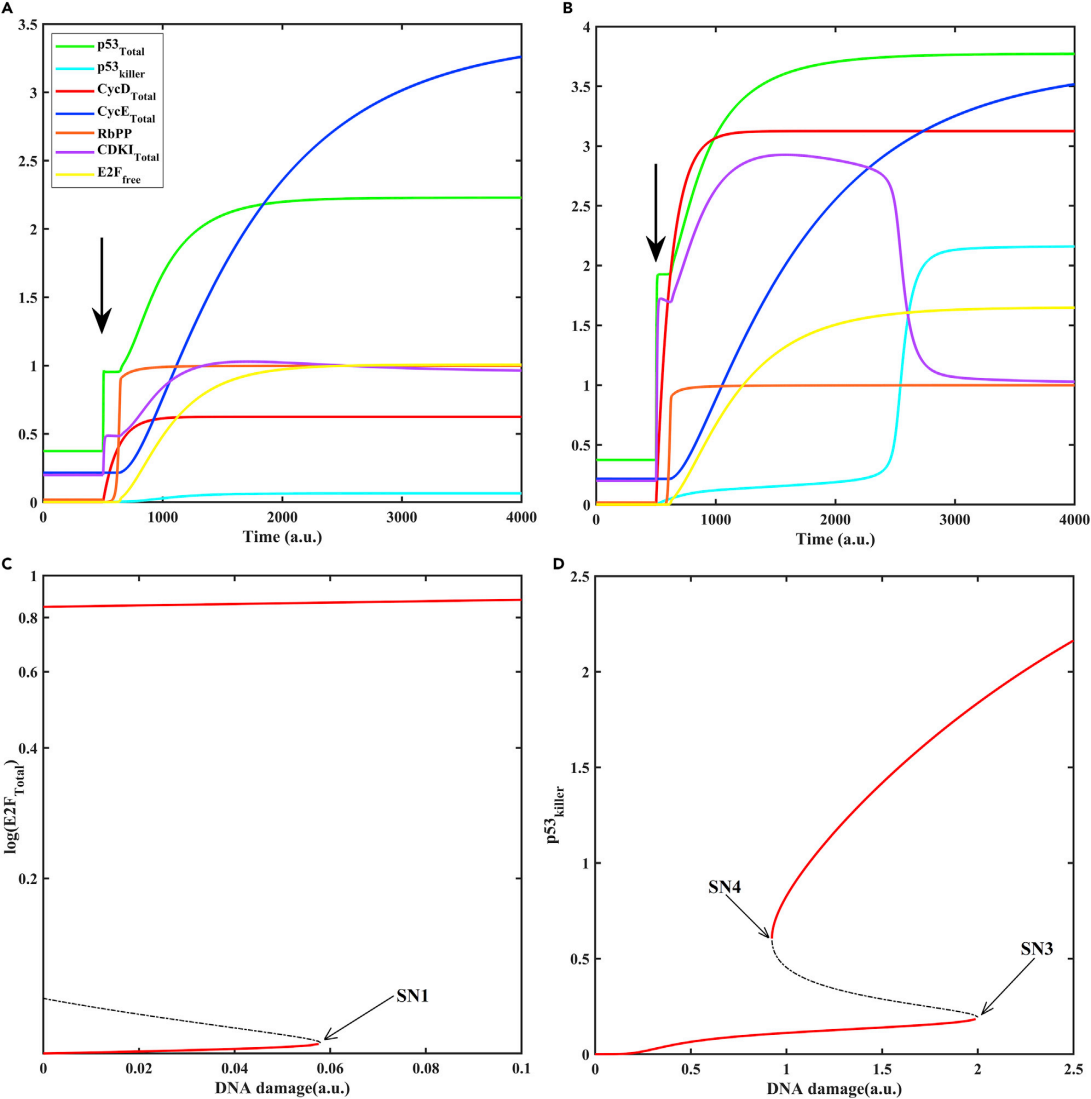
Cell cycle re-entry via DNA damage-dependent E2F activation (A) Temporal dynamics of state variables is shown by setting the DNA damage to intermediate level (0.5 a.u.) from zero at t = 500 (marked by arrow). (B) Temporal dynamics of state variables is shown for higher level of DNA damage (2.5 a.u.) at t = 500 (marked by arrow). (C) The bifurcation diagram showing the bistable activation of E2F for lower values of DNA damage (shown in semilog scale for better resolution). (D) Bistable activation of p53_killer_ for higher values of DNA damage. Solid lines represent stable steady states, while dashed line represents unstable steady state. SN1, SN3, and SN4 represent saddle node corresponding to E2F activation, p53 killer activation, and inactivation, respectively. a.u. represents arbitrary units.

Analogous to mitogen, DNA damage induces the nuclear activity of CycD-Cdk4/6. DNA damage also increases the half-life of E2F by bringing down the degradation rate. However, DNA damage simultaneously induces the expression of p53 helper and its downstream target p21. At a lower extent of DNA damage, the cells remain arrested since the CDKI barrier exceeds total Cdk activity despite an increase in CycD-Cdk4/6, and E2F levels do not exceed the Rb level. At an intermediate level of DNA damage (Figure 8A), Rb is hyperphosphorylated, and E2F attains a higher steady-state value since DNA damage-induced rise in CycD-Cdk4/6 helps cyclins (CycD_Total_ + CycE_Total_) overcome the CDKI barrier (CDKI_Total_). Thus, the relative abundance of activators and inhibitors (cyclins and CDKI; E2F and Rb) determines the cellular state. A higher level of DNA damage (Figure 8B) leads to the accumulation of p53 killer (p53_killer_) that, together with E2F, can induce the expression of apoptotic proteins. The DNA damage-dependent module dynamics captures the observations compiled in Table S3 (Castillo et al., 2015; Shats et al., 2017; Walton et al., 2019; Zhang et al., 2020).

Bifurcation analysis with respect to variation in the level of DNA damage shows a separation of thresholds for Rb inactivation by hyperphosphorylation (E2F activation) (Figure 8C) and p53 killer activation (Figure 8D). Rb gets inactivated at a lower threshold, while p53 killer gets activated at a higher threshold level of DNA damage. At an intermediate level between the two thresholds, the activation of E2F may indicate its functional role in DNA repair, as shown in Figure S2 (Shats et al., 2017). The activation of E2F is irreversible with respect to DNA damage (Figure 8C) due to the feedback loop regulation involving Rb and E2F (Rb --| E2F → CycE-Cdk2 --| Rb). This suggests that once neurons commit to cell cycle re-entry, then there is no point-of-return and may undergo polyploidization (Nandakumar et al., 2021). p53 killer activation shows bistable activation at a higher level of DNA damage (Figure 8D). The activation of p53 killer depends on the activation of the positive feedback loop via p53DINP1, which promotes the conversion between p53 helper to killer. p53DINP1 levels begin to rise initially due to the rise in E2F levels, which, together with p53 killer, amplifies its levels further. Because E2F levels required to activate cell cycle genes differ from the activation of pro-apoptotic genes, the two bistable switches are separated, creating two different thresholds (Figures 8C and 8D). This emergent dynamics delays the activation of apoptosis genes until DNA damage accumulates in neurons to higher levels. An increase in E2F stabilization by DNA damage (fac2, see Methods S3) shifts the saddle nodes of p53 killer activation to lower DNA damage levels reducing the delay in activation of apoptosis (Figure S3). Thus, the graded increase in E2F levels in neurons controls the cell fate decision (Shats et al., 2017).

### Sensitivity of models to parametric variation

We presented a set of models that draw an integrated picture across various experimental observations. The set of parameters used in this study is one such example that describes the physiological and pathological phenotypes. Thus, we tested our choice of parameters by evaluating the model sensitivity after varying each parameter in a 10% increase and decrease range. We quantified alteration in the bifurcation diagram by computing the fold change in threshold value (location of saddle node) with variation in the parameter values for each module (Figures S4–S6). 132 out of the 136 total parameters tested from the three modules showed less than 2-fold change in threshold value. Four parameters viz CKI_T_, Cdk5_T_, k_ierk_, and k_aerk_ (see Methods S1) that directly control the competition between p35 and CycD in module 1 show more than 2-fold change in threshold values in both directions on parameter variation. The irreversible characteristic of the transition from normal to pathological state is preserved for all parameters under these perturbations in module 2 and module 3.

### Expression profile of cell cycle and redox regulators in the AD brain and transgenic mouse model of AD

The experimental data used in this study mostly represent characteristics of different neuronal cell lines. Therefore, we also analyzed clinical data emerging from patients with AD. We studied the transcriptional changes associated with the model outcome in the hippocampus and entorhinal cortex (EC) regions of the AD brain. The beginning of memory loss and cognitive dysfunction is linked to neurodegeneration in EC and hippocampus (Dong et al., 2022; Maruszak and Thuret, 2014; Mu and Gage, 2011; Scharfman and Chao, 2013). The eigengene expression profile (Alter et al., 2000) of cell cycle genes under the transcriptional control of E2F shows a positive correlation with AD compared to normal samples in the EC and hippocampal regions (Table 1). Furthermore, we analyzed the expression pattern of p53 activated pro-apoptotic genes such as Noxa, Bax, p53, p73, p53DINP1, Apaf1, Casp6, p21, and Mdm2 (Fischer, 2017). The eigengene expression profile of these genes also shows a positive correlation with the disease state (Table 1). Additionally, E2F and p53 transcriptional dysregulation is observed in the transgenic mouse model of AD (rTg4510) (Table 1). These findings are consistent with immunoblotting observations showing the activation of p53 in the AD brain (De La Monte et al., 1997; Hooper et al., 2007). p53 is also activated in the Tg2576 transgenic mouse model of AD and with soluble Aβ treatment (Ohyagi et al., 2005). p53 expression in neurons is accompanied by DNA fragmentation (LaFerla et al., 1996). Neurons from different brain regions of the 3x-transgenic AD mice show colocalization of Rb hyperphosphorylation (E2F activation) with a tau pathology marker. Furthermore, the appearance of Rb hyperphosphorylation precedes the appearance of the tau pathology markers in the hippocampus of the AD brain (Hradek et al., 2015).

**Table 1:** Cell cycle and redox metabolism gene expression pattern in AD and GBM

Disease	Region/Group	Gene set	Pearson correlation coefficient	p -value	Identifier
AD	Hippocampus	E2F target	0.306	2.4e-4	GSE28146, GSE29378, GSE36980, GSE48350, GSE5281
		p53 target	0.381	3.4e-6	
		Redox metabolism	−0.359	1.3e-5	
	Entorhinal cortex	E2F target	0.367	9.6e-4	GSE26927, GSE26972, GSE48350, GSE5281
		p53 target	0.35	1.7e-3	
		Redox metabolism	−0.404	2.5e-4	
	Hippocampus	E2F target	0.459	9.4e-3	GSE1297
		p53 target	0.518	2.8e-3	
		Redox metabolism	−0.517	2.9e-3	
	Entorhinal cortex	E2F target	0.718	9.3e-17	GSE118553
		p53 target	0.742	2.2e-18	
		Redox metabolism	0.465	1.5e-6	
Transgenic mouse model of AD	Entorhinal cortex	E2F target	0.372^a^	3.7e-3^a^	GSE125957
		p53 target	0.548^a^	7.0e-6^a^	
		Redox metabolism	0.533^a^	1.4e-5^a^	
		E2F target	0.731^b^	6.6e-6^b^	
		p53 target	0.865^b^	1.4e-9^b^	
		Redox metabolism	0.789^b^	3.8e-7^b^	
GBM	Group 1	p53 target	−0.823	9.2e-5	GSE119834
		Redox metabolism	0.905	1.5e-06	
	Group 2	p53 target	−0.941	5.6e-7	
		Redox metabolism	−0.669	0.009	

Pearson correlation coefficient and corresponding Student asymptotic p value for eigen gene expression profile with disease is given.

a represents correlation of expression profile between wild type and transgenic mice.

b represents correlation of expression profile with transgenic mice age.

Loss of APC/C-Cdh1 function disturbs the balance between pro-oxidants and antioxidants leading to perturbation of redox homeostasis and oxidative stress. Analysis of eigengene expression profile with genes involved in redox metabolism shows a significant dysregulation (Table 1). Most of the AD brain datasets from EC and hippocampus show a negative correlation with the pathological state. However, we also found evidence for a positive correlation with redox metabolism genes in the rTg4510 transgenic mouse model of AD and in one of the AD brain datasets from the EC region (Table 1). The upregulation of redox metabolism may result from a stress-responsive compensatory mechanism. NFT performs an alternative function via induction of a secondary neuroprotective mechanism (Chen and Zhong, 2014; Haque et al., 2019; Lee et al., 2005; Petersen et al., 2007). The expression pattern of genes controlled by E2F, p53, and redox metabolism genes is in accordance with the different states captured by our models. Upregulation of E2F and p53 target genes in AD points toward probable apoptosis signaling, whereas downregulation of redox metabolism gene suggests oxidative stress. This analysis provides supporting evidence in patients confirming these as relevant role players in AD pathogenesis.

### Neurodegeneration versus cancer

Dysregulation of the cell cycle marks the pathological state of both AD and cancers, but neurons die while attempting to divide, whereas cancer cells continue to divide uncontrollably (Roe et al., 2005). Similar to the expression analysis in AD, we studied eigengene expression of p53-activated genes and redox metabolism genes in glioblastoma multiforme (GBM) to understand the underlying molecular difference (Mack et al., 2019; Seo and Park, 2019). Glioblastoma stem cells (GSC) derived from primary tumors contrasted strikingly from their progenitor neural stem cells (NSC) in the expression of p53-activated genes (Table 1). Unlike AD, p53 target genes showed significant downregulation in GBM. p53 gene mutations are frequently observed in different cancers (Breijyeh and Karaman, 2020; Halazonetis et al., 2008). On the contrary, the redox metabolism genes showed heterogeneity across two GBM groups. The group with classical, proliferative features showed upregulation (Pearson Correlation: 0.905; p value: 1.5e-06), whereas the second group with mesenchymal features exhibited downregulation of redox metabolism (Pearson Correlation: −0.669; p value: 0.009). These differences attribute to differences in metabolic reprogramming among the subgroups (Luo and Wicha, 2019).

## Discussion

Cell division plays an important role in tissue regeneration and development. However, unlike most of the other cell types, differentiated neurons are perceived to have entered a permanent postmitotic quiescent state. In AD, neurons undergo atrophy, and this loss is often associated with cell cycle re-activation. We studied the different routes to cell cycle re-entry in postmitotic neurons. The emergent properties of cell cycle control networks were analyzed using a mathematical modeling approach. We show how multiple feedback loops combine to make the transition from normal to pathological state irreversible and explore the effect of different perturbations that provide insights into drug targeting strategies.

The first network module focused on Aβ induced positive feedback between ERK and CycD, which promotes a switch-like activation of ERK activity in neurons. We speculate that Aβ induces dissociation of p35-Cdk5-p27 nuclear complex and translocation of p27 leads to the activation of ERK by eliminating the competition between CycD and p35 for Cdk5. Furthermore, in the resting neurons, the p35-Cdk5-p27 nuclear complex suppresses expression of proliferation promoting E2F1-DP1 target genes by competing with DP1 for E2F1 binding (J. Zhang et al., 2010b). p27 also plays a role in stabilizing the CycD and Cdk4/6 interaction (Xia et al., 2019). An increase in CycD accumulation with ERK activation can lead to an increase in CycD-Cdk4/6 activity, which influences the Rb-E2F switch by monophosphorylating Rb. CycD-Cdk4/6 derepresses genes under the control of RbL2/p130-E2F4 complex by phosphorylation. RbL2/p130-E2F4 is known to suppress the pro-apoptotic gene B-Myb in healthy neurons (Greene et al., 2007). This complex also participates in the formation of the DREAM complex, which suppresses MMB-FOXM1 target genes, including CycB (Fischer et al., 2016). In addition, ERK also controls cell cycle progression by regulating Cdk2 location and Cdk1 activity (Chambard et al., 2007; Ding et al., 2000; Leung et al., 2008). CycB-Cdk1 is known to trigger phosphorylation of pro-apoptotic proteins BAD and FOXO1. In the absence of Akt signaling, CycB-Cdk1-dependent phosphorylation relieves the inhibition of these proteins by scaffold protein 14-3-3, leading to apoptosis in neurons (Brunet et al., 2001; Castedo et al., 2002; Konishi et al., 2002; Yuan et al., 2008). Interestingly, Aβ is also known to inhibit Akt activity (Abbott et al., 2008; Lee et al., 2009b).

An alternate route to CRNA centered around APC/C-Cdh1 inactivation mediated by Aβ-dependent Ca^2+^ dysregulation and oxidative stress. We showed an irreversible transition to a high ROS state at higher levels of Aβ due to the multiple feedback regulations of APC/C-Cdh1. The irreversible transition also suggests that decreasing Aβ alone may not have the desired effect. We modeled inhibition of Gls1 and/or CycB that helps to reduce the levels of ROS, resulting in rescue. We observed glutaminase inhibitor completely abolishes the effect of APC/C-Cdh1 inhibitor but not of Aβ (Fuchsberger et al., 2016). On the contrary, MEK inhibition appears to completely rescue CRNA induced by Aβ (Modi et al., 2012), which suggests crosstalk between these network modules and the ERK switch probably acts as an initiator module for apoptosis. We propose a scenario of how these two modules can crosstalk via Cdk5 regulation. The autophosphorylation of p35-Cdk5 protects it from calpain protease activity, but Aβ-dependent rise in the CycD-Cdk5-p27 complex dissociates the p35-Cdk5 complex and makes p35 more susceptible to cleavage (Kamei et al., 2007; Shah and Lahiri, 2014). Hence, CycD induction reduces p35 and Cdk5 association (module 1) and helps in Ca^2+^-dependent generation of p25, which binds Cdk5 strongly compared to CycD (Modi et al., 2012). Subsequently, p25-Cdk5 inhibits APC/C-Cdh1 (module 2) and also phosphorylates substrates nuclear lamin, anti-apoptotic protein Mcl-1, and cytoskeletal proteins that can promote a transition to the apoptotic state (Allnutt et al., 2020; Nikhil and Shah, 2017). These suggest that cell cycle re-entry may activate multiple routes to apoptosis. However, Aβ-induced cell cycle re-entry also protects some proportion of neurons from apoptosis (Ippati et al., 2021). This indicates that cell fate decisions may be influenced by the heterogeneity in the stress levels (oxidative stress and DNA damage) experienced by individual neurons.

The third passage to CRNA focused on DNA damage-mediated CycD induction and E2F stabilization. Neurons make an irreversible commitment to the cell cycle with an increase in the level of E2F. This may play a role in DNA repair (Stevens and La Thangue, 2004) and polyploidization (Nandakumar et al., 2021) that protects them from cell death under DNA damage and oxidative stress. A further increase in E2F level in a graded manner with an increase in DNA damage may lead to the expression of pro-apoptotic gene expression in cooperation with p53 (Shats et al., 2017; Wu and Levine, 1994). We showed that the different thresholds for activation of DNA repair and apoptosis emerge by combining two bistable switches. The extent of DNA damage can exceed repair threshold if ROS (as observed with module 2) levels rise, which can induce oxidative DNA damage.

In our previous study, we captured the dynamics of how stress signals like oxidative stress and DNA damage induced transition back to quiescence after crossing the restriction point and before the G1/S boundary in mammary epithelial cell line (Pandey and Vinod, 2018). In contrast, we observed a scenario of how stress signals drive cell cycle re-entry in neurons. We attribute this difference to the defense mechanism in neurons against DNA damage since the levels of repair proteins are very low in the mature neuron state (Kruman et al., 2004; Schwartz et al., 2007). This view differs from the recently proposed “cell cycle inertia” driven mechanism for the G1/S transition in the presence of stress signals close to the G1/S boundary in mammary epithelial cells. Here, cells commit to S-phase due to inertia from rising Cdk2 activity with a slower accumulation of CDKI (Nathans et al., 2021).

The pre-clinical phase of AD is characterized by neurons entering the cell cycle. The irreversible nature of AD points toward the need for understanding the disease progression mechanism in greater detail. The failure of therapeutic measures at various phases of clinical trials reflects mere removal of the causative agent is not sufficient for efficient treatment. We showed the convergence of cell cycle re-entry onto the activation of self-amplifying positive feedback loops, and shutting off feedback signals may serve as an efficient disease-modifying therapy. We also proposed mechanisms through which crosstalk between different routes to CRNA may take place and compared the scenario with cell cycle progression in other mammalian cells.

### Limitations of the study

The work presented here investigated the network modules that set off CRNA and studied the role of different feedback loops in pushing the system into an irrevocable pathological state. The mathematical models were developed to simulate the multiple scenarios for cell cycle re-entry with a minimal number of state variables. Hence, the role of some cell cycle regulators was not explicitly modeled since they converge on the network presented here. For instance, miR26b levels are found to be high in AD and are known to control the Rb-E2F switch by inhibiting the Rb transcripts (Absalon et al., 2013). The expression of DTL, an E2F target, was upregulated in AD across multiple brain regions, including the EC (Fischer et al., 2016; Huang et al., 2019). It downregulates p21, thereby promoting Cdk activity. Because the underlying mechanism is similar to CycE-dependent regulation of p21 (module 3), the DTL effect was not separately modeled. It can be noted that E2F activation also depends upon other stress kinases like p38^MAPK^ (Frade and Ovejero-Benito, 2015; Morillo et al., 2012). Different kinases also regulate cytoplasmic localization of p27. Furthermore, cytoplasmic p27 controls autophagy, which aids the clearance of protein aggregates and delays apoptosis (Dong et al., 2022). Because the Aβ clearance therapies fail to bring about disease reversal, the focus of our study was on self-amplifying positive feedback loops. Our models capture the Aβ-induced CRNA scenarios but do not account for the cell cycle-independent effects. While Aβ was an input in our models, Aβ amplification itself due to feedback loop regulation can happen in AD (Doig, 2018). We speculate that depending on the order of events additional feedback loops may bring down the threshold (saddle node) for transition to the pathological state and push the neurons into a deeper state of irreversibility.

## STAR★Methods

### Key resources table

**Table:** 

REAGENT or RESOURCE	SOURCE	IDENTIFIER
**Deposited data**		

Postmortem AD brain samples	http://alzdata.org/download1.php Cross platform normalized dataset of Hippocampus	GEO: GSE28146, GSE29378, GSE36980, GSE48350, GSE5281
Postmortem AD brain samples	http://alzdata.org/download1.php Cross platform normalized dataset of Entorhinal cortex	GEO: GSE26927, GSE26972, GSE48350, GSE5281
Postmortem AD brain sample	https://www.ncbi.nlm.nih.gov/geo/	GEO: GSE1297
Postmortem AD brain samples	https://www.ncbi.nlm.nih.gov/geo/	GEO: GSE118553
Transgenic mouse rTg4510 model organism	http://www.epigenomicslab.com/ADmice/ Tg4510 normalised counts	GEO: GSE125957
Neural stem cells	https://www.ncbi.nlm.nih.gov/geo/	GEO: GSE119834
Glioblastoma stem cells		

**Software and algorithms**		

Matlab 2019a	https://in.mathworks.com/products/matlab.html	NA
XPPAUT	http://www.math.pitt.edu/∼bard/xpp/xpp.html	NA
R for windows (4.1.2)	https://cran.r-project.org/bin/windows/base/	NA
RStudio v2021.09.0+351	https://www.rstudio.com/products/rstudio/download/#download	NA
biomaRt v2.50.3	https://bioconductor.org/packages/release/bioc/html/biomaRt.html	NA
GEOquery v2.62.2	https://bioconductor.org/packages/release/bioc/html/GEOquery.html	NA
WGCNA 1.70-3	https://horvath.genetics.ucla.edu/html/CoexpressionNetwork/Rpackages/WGCNA/	NA
preprocessCore v1.56.0	http://www.bioconductor.org/packages/release/bioc/html/preprocessCore.html	NA

### Resource availability

#### Lead contact

Further information should be directed to and will be fulfilled by the lead contact, Vinod P.K. (vinod.pk@iiit.ac.in).

#### Materials availability

This study did not generate new unique reagents.

### Method details

#### Translation of molecular networks into mathematical models

The three modules capturing alternative routes to cell cycle re-entry are regulated by complex molecular networks. These network modules presented in Figure 1, Figure 2, Figure 3 were translated into a set of ordinary differential equations (ODE) and algebraic equations to describe the dynamics of individual components. Unless stated otherwise, the law of mass action was used to represent the synthesis, degradation, activation, inactivation, association, dissociation reactions, and transport mechanisms.

The equations and parameters values corresponding to module 1 is given in Methods S1. Experimental evidence suggests that the ERK total protein levels don’t change, but Aβ stimulation alters its activity via MEK-1 dependent phosphorylation of ERK (Modi et al., 2012). Hence, the ERK total was modeled as a fixed parameter and its activity was considered to be directly controlled by Aβ. The activation/inactivation of ERK, which is known to exhibit ultrasensitive characteristics, was modeled as Michaelis-Menten kinetics (Equation 8) (Goldbeter and Koshland, 1981; Huang and Ferrell, 1996). Considering the physiological function and ubiquitous abundance of p35, Cdk5 and p27 in differentiated neurons, the total concentration of p35 (p35_T_), Cdk5 (Cdk5_T_) and p27 (CDKI_T_) were modeled as a fixed parameter in module 1.

The equations and parameters values corresponding to module 2 is given in Methods S2. Rb and APC/C-Cdh1 activity is controlled by p25-Cdk5 and CycB-Cdk1 dependent phosphorylation, with their total levels fixed. The activation/inactivation of Rb (Equations 12 and 13) and APC/C-Cdh1 (Equation 4) was modeled as Michaelis-Menten kinetics and E2F dependent synthesis of E2F (autoactivation) and CycB (Equations 6 and 11) were modeled as Hill function. The model equations and parameters used to describe these variables were taken from Pandey and Vinod (2018) and is provided in Methods S2. Ca^2+^ dependent activation of calpain in module 2 was also modeled as Hill function (Equation 2) since cooperative binding of two Ca^2+^ ions to calpain is known (Moldoveanu et al., 2002). Similar to module 1, p35 was considered a fixed parameter. The activity of Cdk (Cdk5, Cdk1) was considered to be limited by its binding partner. Hence, the variables p35, p25, CycB represent the corresponding Cdk activity. We considered direct regulation of NADPH by APC/C-Cdh1, CycB regulation by E2F, ROS regulation by CycB, Ca^2+^ and ROS mutual amplification eliminating the intermediate steps involved in these regulations (see Module 2 description) to keep the model minimalistic.

The equations and parameters values corresponding to module 3 is given in Methods S3. Rb-E2F regulation is modelled similar to module 2. In the E2F-p53 coordinated apoptotic signaling, p53DINP1 (Equation 7) and p53 killer synthesis (Equation 3) were modeled as Hill function (Pandey and Vinod, 2018; Zhang et al., 2009; X. P. Zhang et al., 2010). The cyclin (CycD, CycE) levels control the corresponding Cdk activity (Cdk4, Cdk2).

Aβ was varied as the input parameter in module 1 and 2 to simulate the pathological state. The translocation of p27 into the cytosol (Methods S1) and levels of Ca^2+^ (Methods S2) were regulated by Aβ. DNA damage was the stimulus for module 3. The degradation rates of p53 and E2F were reduced, while that of Mdm2 was increased in a DNA damage dependent manner (Methods S3). The synthesis rate of CycD was increased in the presence of DNA damage.

The work focused on studying the emergent properties of molecular networks and the various perturbations and rescue experiments listed in the supplemental information (Tables S1–S3) (Castillo et al., 2015; Fuchsberger et al., 2016; Giovanni et al., 1999; Herrero-Mendez et al., 2009; Jaiswal and Sharma, 2017; Modi et al., 2012; Shats et al., 2017; Veas-Pérez De Tudela et al., 2015b, 2015a; Walton et al., 2019; J. Zhang et al., 2010a; Zhang et al., 2020). We have attempted to bring together a variety of *in vitro* perturbation (chemical inhibition, gene knockout, mutations, overexpression) experiments that directly target cell cycle proteins or indirectly affect cell cycle regulatory proteins under various input conditions, including Aβ treatment and DNA damage. The experimental data indicates consistency in the cellular response across the same set of stimuli and inhibitors, but the quantitative measure showed differences. These variations may arise due to the differences in experimental handling, intrinsic noise, differences in cell line, etc. Therefore, we combined these observations to present qualitative models that draw a consensus across multiple studies.

We started the model simulations with parameter values obtained from cell cycle and apoptosis models (Pandey and Vinod, 2018; Zhang et al., 2009; X. P. Zhang et al., 2010). The models were integrated, and the parameter values were refined to simulate the data corresponding to Aβ and DNA damage induced cell cycle re-entry and apoptosis (Methods S2 and S3). The initial levels/activity and cellular localization of variables representing differentiated states were defined based on observations from neuronal cell line experiments (Akashiba et al., 2006; Almeida, 2012; Chen et al., 2012; Gieffers et al., 1999; Jaiswal and Sharma, 2017; Sumrejkanchanakij et al., 2003; Yan and Ziff, 1995; J. Zhang et al., 2010b, 2010a). The *in vitro* experiments were used to calibrate the model to be consistent with the qualitative observation of cell cycle re-entry or apoptosis under various perturbations (Tables S1–S3). In addition to Aβ, glutamate excitotoxicity, APC/C-Cdh1 inactivation and CycB-Cdk1 overexpression were used to model the alternate triggers for cell cycle re-entry. We also calibrated the model to distinguish the thresholds for DNA damage induced repair and CRNA.

The complex formation is assumed to be rapid compared to synthesis, degradation, activation, inactivation, and transport rate constants. The knockout (KO)/inhibition experiments for state variables were simulated by setting either the synthesis or activation rate to zero; for fixed parameter, the KO condition was modeled by setting the total level of protein to zero. One and two-parameter bifurcation analyses were performed using XPPAUT to characterize how the system responds to variations in the parameter values and study the effect of individual feedback loops. The default parameters were also varied in a 10% increase and decrease range to test parameter sensitivity. The set of equations were solved numerically with XPPAUT (http://www.math.pitt.edu/∼bard/xpp/xpp.html).

The simulations represent a dynamic picture but not the actual time scale of disease progression due to the unavailability of temporal data on systemic changes in disease progression. Therefore, rate constants (k) have a dimension of time^−1^. The state variables represent relative concentrations of respective components and are dimensionless. Michaelis constants (J) and half-saturation constants are also dimensionless. The complete model, including equations, parameters, state variables, and XPPAUT code, are provided as supplemental information.

#### Analysis of transcriptome data from AD patients and transgenic animal model

Different model scenarios for cell cycle re-entry in neurons may lead to transcriptional changes. Transcriptomic data of AD were analyzed to study the model predicted changes in gene expression related to the proposed modules. Normalized expression data across multiple datasets from different regions of AD brain postmortem samples and control samples were retrieved from http://www.alzdata.org/(Xu et al., 2018; Zhang et al., 2019). Additional AD brain datasets were downloaded from gene expression omnibus (GEO) and processed using the GEO2R R script (Barrett et al., 2013; Blalock et al., 2004; Edgar et al., 2002; Patel et al., 2019; Sean and Meltzer, 2007). These datasets resolve samples into different groups: asymptomatic, incipient, moderate, and severe AD. Further, normalized temporal data from the rTg4510 transgenic mouse model was also used to study progressive changes in AD (Castanho et al., 2020). The key resource table summarizes details of the dataset used.

The gene expression pattern of various targets of E2F, p53, and redox metabolism were studied. The list of genes under the regulation of these transcriptional factors and redox metabolism were obtained from the literature (Data S1) (Benfeitas et al., 2019; Fischer, 2017; Fischer et al., 2016). E2F target genes which were associated with the cell cycle in at least one study were filtered (Fischer et al., 2016). The eigengene expression profile representing the maximum variance for the groups of genes of interest (E2F target, p53 target, and redox metabolism) were obtained for each sample using the *moduleEigengenes* function of the weighted correlation network analysis (WGCNA) package in *R* (Langfelder and Horvath, 2008). Correlation between the representative eigengene expression and disease state was obtained by the Pearson correlation method using the *cor* function in *R*.

Since both neurodegeneration and cancer show aberrant cell cycle re-entry, our results from AD were also compared with the expression patterns from cancer. The transcriptomic data from primary tumor-derived GSC and NSC was analyzed (Mack et al., 2019). The FPKM (Fragments Per Kilobase of transcript per Million mapped reads) data was quantile normalized using the *normalize.quantiles* function of preprocessCore package and then log transformed. GSC samples were subdivided into two distinct groups based on the classification provided by Mack et al. (2019). The eigengene expression under these conditions was also calculated to study the correlation with disease.

### Quantification and statistical analysis

Statistical significance of the correlation values was obtained using the *corPvalueStudent* function in R that computes Student asymptotic p value for given correlations (Langfelder and Horvath, 2008).

## Data Availability

• This paper analyzes existing, publicly available data. These accession numbers for the datasets are listed in the key resources table. • All original code is available in this paper’s supplemental information (XPPAUT code for cell cycle re-entry module 1, module 2, module 3). • Any additional information required to reanalyze the data reported in this paper is available from the lead contact upon request.

## Supplemental information

Document S1. Figure S1–S6 and Table S1–S3

Methods S1. Mathematical model, parameter values and dynamic variables of module 1, related to STAR Methods

Methods S2. Mathematical model, parameter values and dynamic variables of module 2, related to STAR Methods

Methods S3. Mathematical model, parameter values and dynamic variables of module 3, related to STAR Methods

Data S1. List of genes under the regulation of E2F, p53 and redox metabolism genes used for expression data analysis, related to STAR Methods
