# Supplementary material for: Model scenarios for cell cycle re-entry in Alzheimer's disease
Source: iScience. 2022 Jun 7;25(7):104543. doi: 10.1016/j.isci.2022.104543 (PMC9209725; doi:10.1016/j.isci.2022.104543)
Supplement: Document S1. Figure S1–S6 and Table S1–S3 [file mmc1.pdf]

iScience, Volume *25*

Supplemental information

Model scenarios for cell cycle re-entry in Alzheimer's disease

Nishtha Pandey and P.K. Vinod

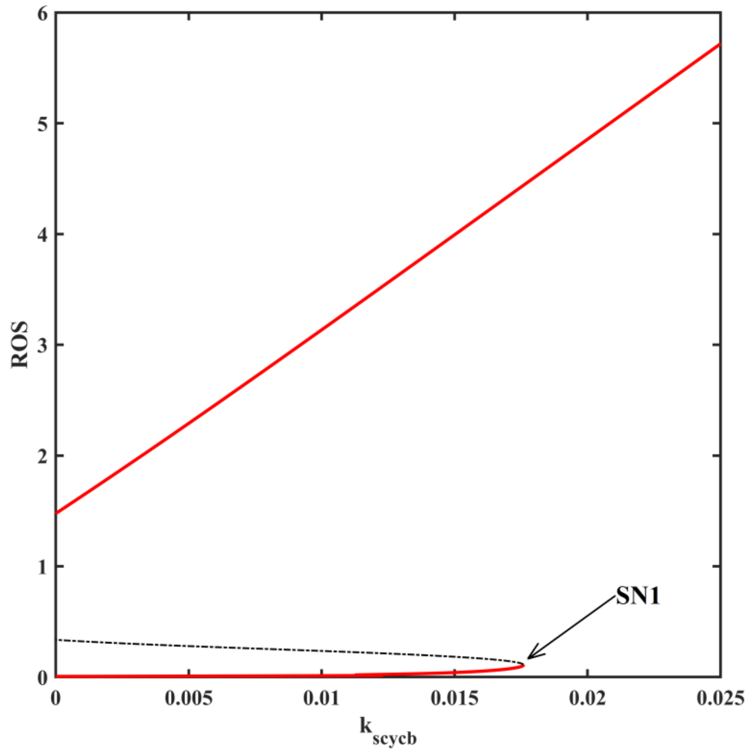

Figure S1. The bifurcation diagram showing the effect of CycB overexpression (k_{scycb}) on ROS accumulation. The rise in CycB can drive the transition to irreversible pathological state, related to Figure 7.

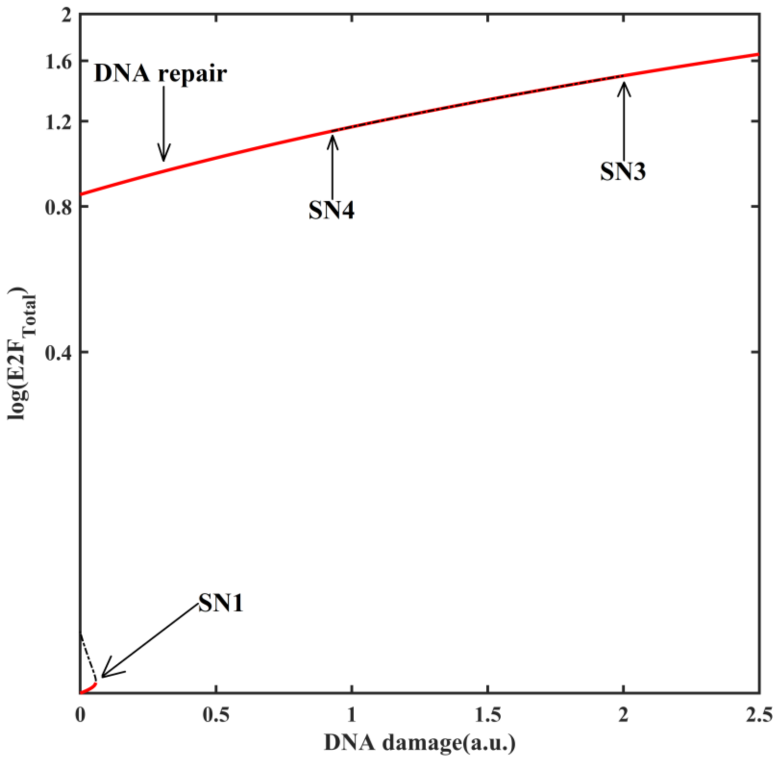

Figure S2. The bifurcation diagram showing the variation in E2F levels for lower and higher DNA damage levels. E2F levels corresponding to an intermediate level of DNA damage (above SN1 but below SN3) may play a role in DNA repair. SN1 and SN3 correspond to DNA damage threshold for E2F and p53 killer activation, related to Figure 8c and 8d, respectively.

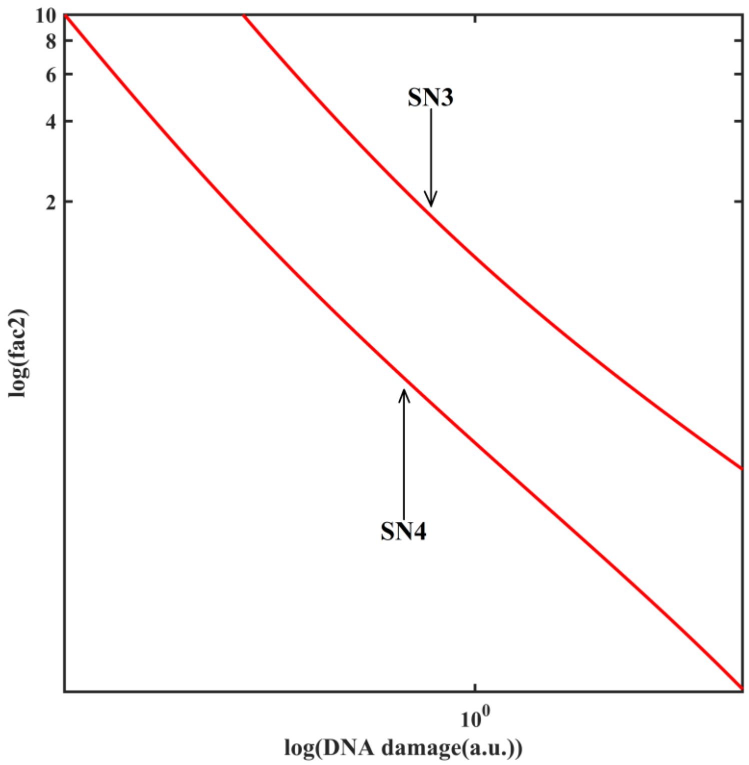

Figure S3. The two-parameter bifurcation analysis showing the effect of altering the stability of E2F (fac2) on the DNA damage threshold for p53 killer activation (SN3) and inactivation (SN4), related to Figure 8d.

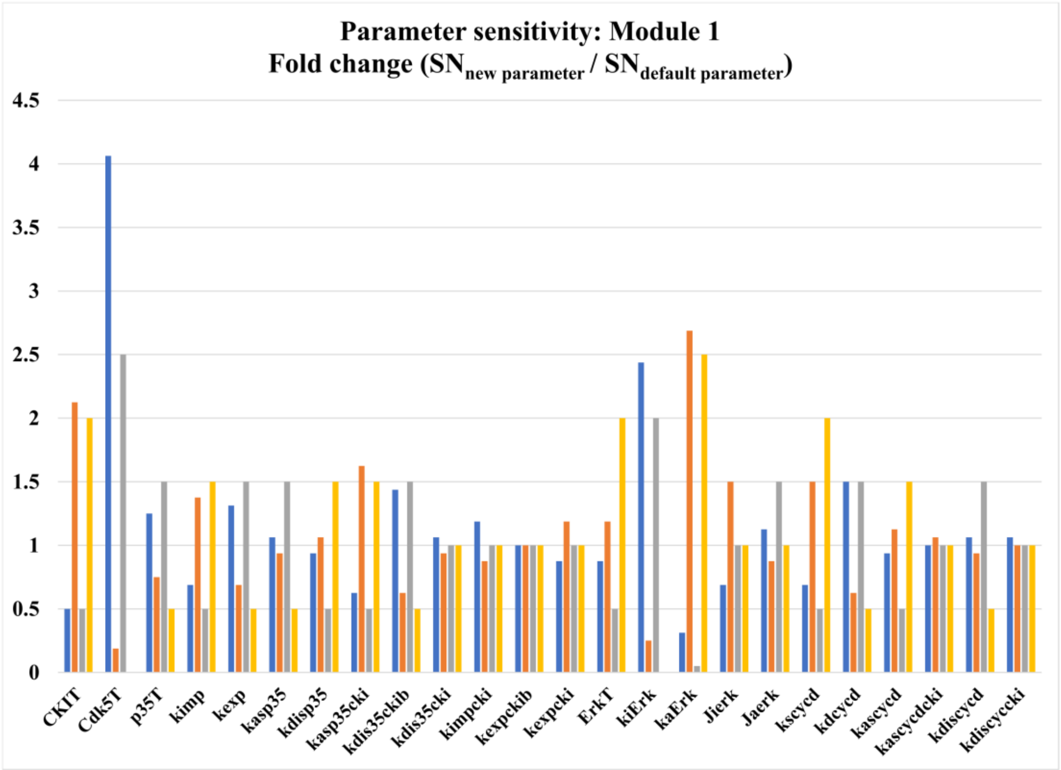

Figure S4. Parameter sensitivity analysis for module 1. The color represents the fold change in saddle node values for change in parameter (increase or decrease) from default values (fold change = SN(new)/SN(default). Fold change in SN1 with increase (blue) and decrease (orange) in parameter values and fold change in SN2 with increase (grey) and decrease (yellow) in parameter values are shown, related to STAR methods.

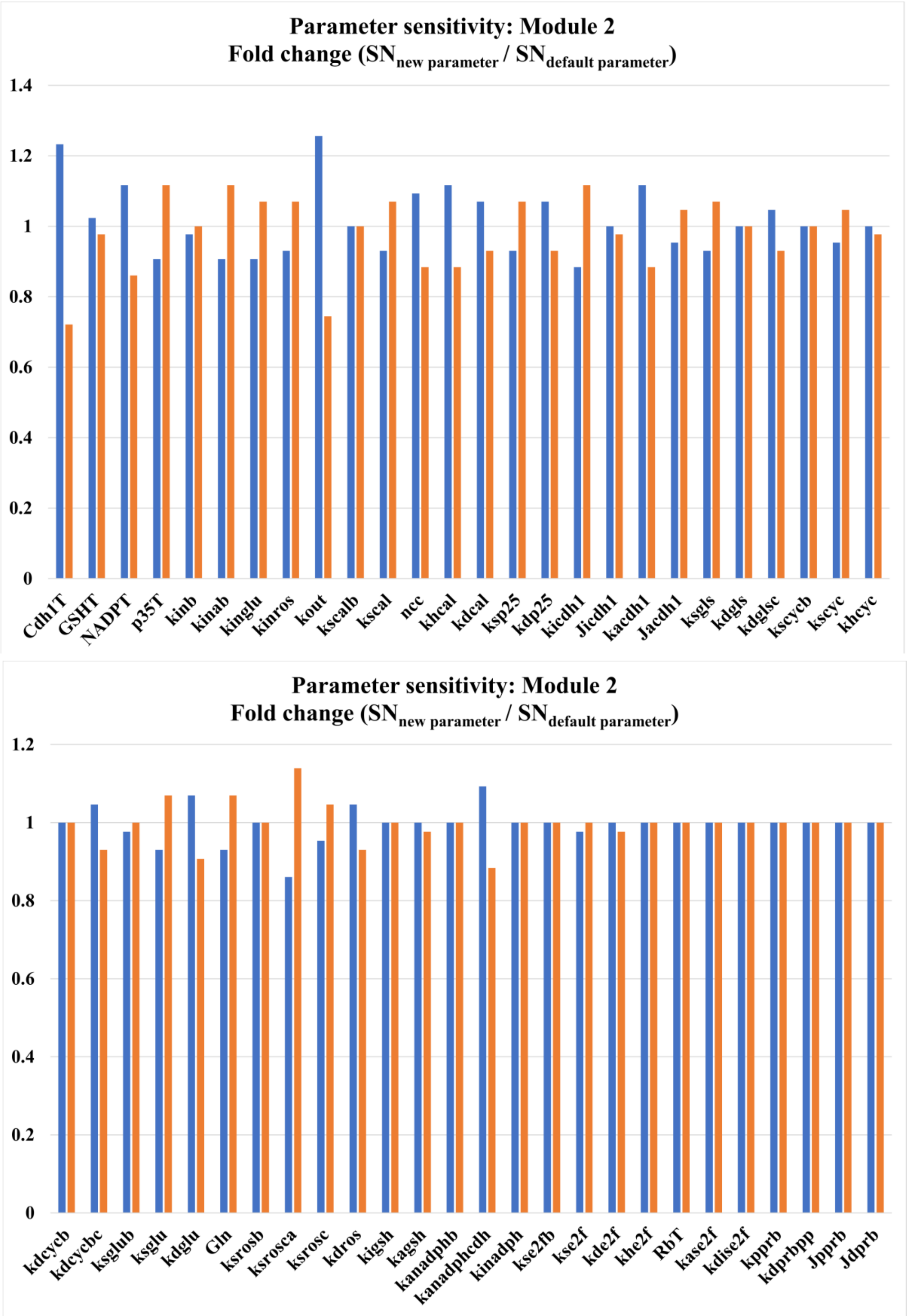

Figure S5. Parameter sensitivity analysis for module 2. The color represents the fold change in saddle node values for change in parameter (increase or decrease) from default values (fold change = SN(new)/SN(default). Fold change in SN1 with increase (blue) and decrease (orange) in parameter values are shown, related to STAR methods.

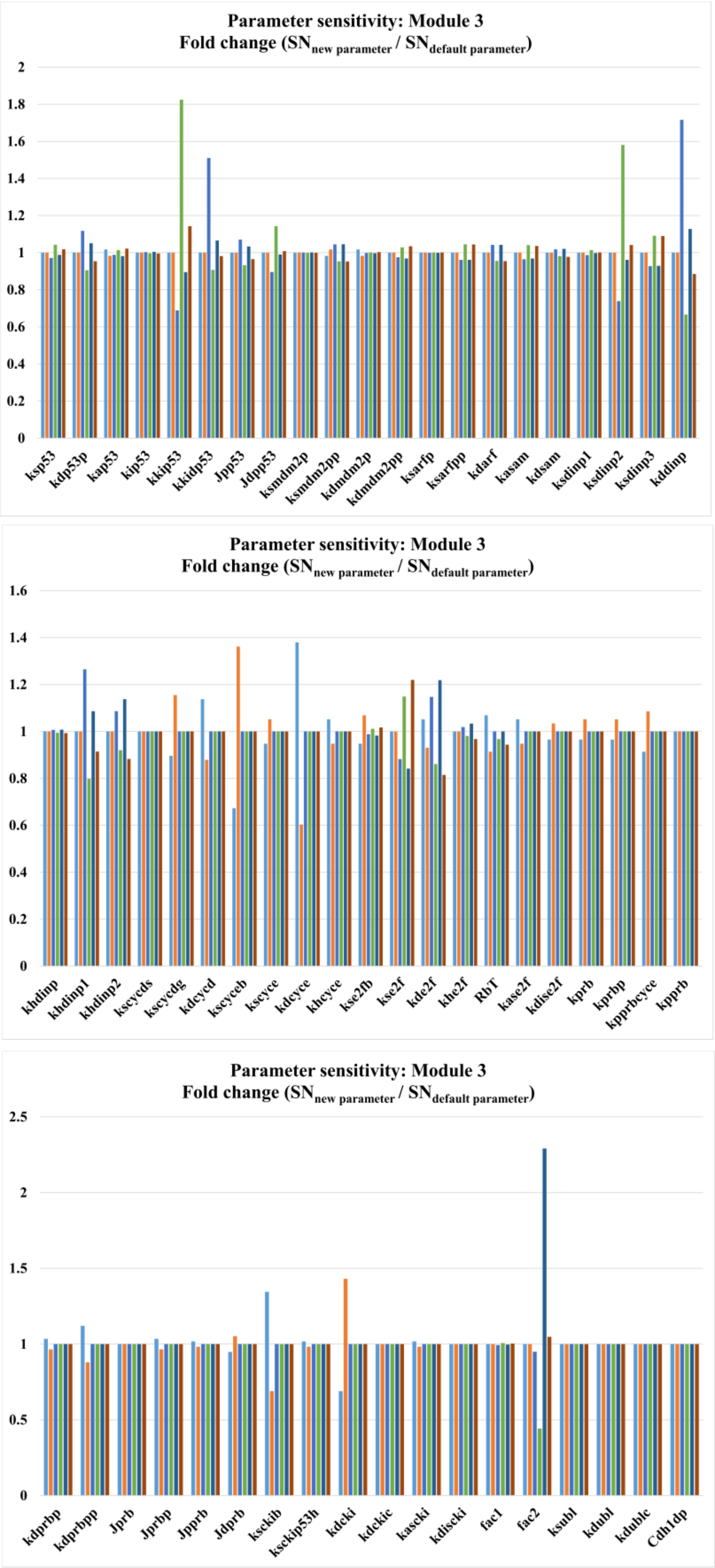

Figure S6. Parameter sensitivity analysis for module 3. The color represents the fold change in saddle node values for change in parameter (increase or decrease) from default values (fold change = SN(new)/SN(default). SN1: increase (blue) and decrease (orange); SN3: increase (light blue) and decrease (green); and SN4: increase (navy blue) and decrease (brown), related to STAR methods.

Cell line	Stimulus	Rescue	Cell cycle entry	Apoptosis	Reference
Primary cortical neuron (Rat)	$A\beta_{42}$	-----------------	Yes	Yes	(Modi et al., 2012)
	$A\beta_{42}$	MEK inhibitor	No	No	
	$A\beta_{42}$	CycD siRNA	No	No	
Primary cortical neuron (Rat)	Soluble $A\beta_{1\text{-}42}$ oligomer	-----------------	Yes	Yes	(Jaiswal and Sharma, 2017)
	Soluble $A\beta_{1\text{-}42}$ oligomer	p27siRNA	Yes	No	
Primary cortical neuron (Rat)	Aβ aggregate	-----------------	-----------------	Yes	(Giovanni et al., 1999)
	Aβ aggregate	Cdk4 inhibition	-----------------	No	
	Aβ aggregate	Cdk6 inhibition	-----------------	No	
Primary cortical neuron (*Cdk5-/-* Mice)	Fibrillar $A\beta_{1-42}$	Cdk5-NLS	No	Yes	(Zhang et al., 2010)
		Cdk5-NES	Yes	No	

Table S1. Summary of experimental data used to develop the framework of module 1, related to STAR methods.

<table>
<tr><th>Cell line</th><th>Stimulus</th><th>Rescue</th><th>Cell cycle entry (Approx.)</th><th>Apoptosis (Approx.)</th><th>Reference</th></tr>
<tr><td rowspan="7">Primary cortical neuron (Rat)</td><td rowspan="3">Glutamate excitotoxicity</td><td>----------------</td><td>----------------</td><td>Yes</td><td rowspan="7">(Veas-Pérez De Tudela et al., 2015a)</td></tr>
<tr><td>Cyclin B1 inhibition</td><td>----------------</td><td>No</td></tr>
<tr><td>CDK inhibition</td><td>----------------</td><td>No</td></tr>
<tr><td rowspan="4">Cyc B1 expression</td><td>----------------</td><td>----------------</td><td>Yes</td></tr>
<tr><td>CDK inhibition</td><td>----------------</td><td>No</td></tr>
<tr><td>Phospho-defective Bcl-xL expression</td><td>----------------</td><td>No</td></tr>
<tr><td>Phospho-mimetic Bcl-xL expression</td><td>----------------</td><td>Yes</td></tr>
<tr><td rowspan="6">Primary cortical neuron (Rat)</td><td rowspan="6">Glutamate excitotoxicity</td><td>--------------------</td><td>Yes</td><td>Yes</td><td rowspan="6">(Veas-Pérez De Tudela et al., 2015b)</td></tr>
<tr><td>p27 expression</td><td>No</td><td>------------</td></tr>
<tr><td>Cyclin B1 inhibition</td><td>No</td><td>------------</td></tr>
<tr><td>Phospho-mimetic Cdh1 (inactive)</td><td>Yes</td><td>Yes</td></tr>
<tr><td>Phospho-defective Cdh1 (active)</td><td>No</td><td>No</td></tr>
<tr><td>Cdk5 inhibition</td><td>No</td><td>No</td></tr>
<tr><td rowspan="6">Primary cortical neuron (Rat)</td><td rowspan="2">Soluble $A\beta_{1\text{-}42}$ oligomer</td><td>---------------------</td><td colspan="2">Yes</td><td rowspan="6">(Fuchsberger et al., 2016)</td></tr>
<tr><td>Glutaminase inhibitor</td><td colspan="2">No</td></tr>
<tr><td rowspan="2">Glutamate</td><td>---------------------</td><td colspan="2">Yes</td></tr>
<tr><td>Glutaminase inhibitor</td><td colspan="2">No</td></tr>
<tr><td rowspan="2">APC/C-Cdh1 inhibitor</td><td>---------------------</td><td colspan="2">Yes</td></tr>
<tr><td>Glutaminase inhibitor</td><td colspan="2">No</td></tr>
<tr><td rowspan="6">Primary cortical neuron (Rat)</td><td rowspan="4">APC/C-Cdh1 inhibitor</td><td>---------------------</td><td>---------------</td><td>Yes</td><td rowspan="6">(Herrero-Mendez et al., 2009)</td></tr>
<tr><td>Cyc B1 inhibition</td><td>---------------</td><td>No</td></tr>
<tr><td>Pfkfb3 inhibition</td><td>---------------</td><td>No</td></tr>
<tr><td>Cyc B1 + Pfkfb3 inhibition</td><td>---------------</td><td>No</td></tr>
<tr><td rowspan="2">Pfkfb3 expression</td><td>---------------------</td><td>---------------</td><td>Yes</td></tr>
<tr><td>Glutathione</td><td>---------------</td><td>No</td></tr>
</table>

Table S2. Summary of experimental data used to develop the framework of module 2, related to STAR methods.

Cell line	Stimulus	Rescue	Cell cycle entry (Approx.)	Apoptosis (Approx.)	Reference
Primary cortical neuron (Mice)	Camptothecin	-----------------	Yes	Yes	(Zhang et al., 2020)
	E2F1 transfection	-----------------	Yes	Yes	
SH-SY5Y cells	H_2O_2	-----------------	------------	Yes	(Castillo et al., 2015)
	H_2O_2	E2F inhibitor	------------	Yes	
U2OS cells	E2F1 transfection (low)	-----------------	Yes	-------------	(Shats et al., 2017)
	E2F1 transfection (medium)	-----------------	Yes (and DNA repair)	-------------	
	E2F1 transfection (high)	-----------------	Yes	Yes	
Primary hippocampal neurons (Mice)	CycE-Cdk2 transfection	-----------------	Yes	Yes	(Walton et al., 2019)
	CycE-Cdk2 transfection	p53DN (dominant negative)	Yes	No	

Table S3. Summary of experimental data used to develop the framework of module 3, related to STAR methods.

XPPAUT code for cell cycle re-entry module 1 (Aβ-induced hyperactivation of extracellular signal-regulated kinases (ERK) in neurons), related to STAR methods.

```
# Initial conditions for dynamic variables
init p35n=0.016,Cdk5n=0.003,p35Cdk5n=0.027,p35CdkCKIn=0.838
init p35Cdk5c=0.741,p35CdkCKIc=0.023,CKIn=3.056
init ERKI=0.905,CycDT=0.473,CYCDCDK5C=0.266,CYCCDKCKIC=0.08

# Values of kinetic parameter
par Abeta=0.05
par CKIT=4,Cdk5T=2,p35T=2
par kimp=0.4,kexp=10
par kasp35=100,kdisp35=1
par kasp35cki=10,kdis35ckib=1,kdis35cki=10
par kimpcki=10,kexpckib=0.01,kexpcki=10
par ErkT=1
par kierk=0.25, kaerk=0.1, Jierk=0.1, Jaerk=0.1
par kscycdb=0, kscycd=0.01, kdcycd=0.002
par kascycd=10,kascycdcki=10,kdiscycd=0.1,kdiscyccki=0.1

# Set of differential equations:
# p35n represents the concentration of free nuclear p35
p35n' = kdisp35*p35Cdk5n + kimp*p35c - kasp35*p35n*Cdk5n - kexp*p35n

# Cdk5n represents the concentration of free nuclear Cdk5
Cdk5n' = kdisp35*p35Cdk5n + kimp*Cdk5c - kasp35*p35n*Cdk5n - kexp*Cdk5n

# p35Cdk5n represents the concentration of nuclear p35-Cdk5 complex
p35Cdk5n' = kasp35*p35n*Cdk5n + (kdis35ckib + kdis35cki*Abeta)*p35CdkCKIn +
kimp*p35Cdk5c - kdisp35*p35Cdk5n - kasp35cki*p35Cdk5n*CKIn - kexp*p35Cdk5n

# p35CdkCKIn represents the concentration of nuclear p35-Cdk5-CDKI complex
p35CdkCKIn' = kasp35cki*p35Cdk5n*CKIn - (kdis35ckib +
kdis35cki*Abeta)*p35CdkCKIn

# p35Cdk5c represents the concentration of cytoplasmic p35-Cdk5 complex
p35Cdk5c' = kasp35*p35c*Cdk5c + (kdis35ckib + kdis35cki*Abeta)*p35CdkCKIc +
kexp*p35Cdk5n - kdisp35*p35Cdk5c - kasp35cki*p35Cdk5c*CKIc - kimp*p35Cdk5c

# p35CdkCKIc represents the concentration of cytoplasmic p35-Cdk5-CDKI complex
p35CdkCKIc' = kasp35cki*p35Cdk5c*CKIc - (kdis35ckib + kdis35cki*Abeta)*p35CdkCKIc

# CKIn represents the concentration of free nuclear CDKI
CKIn' = kimpcki*CKIc + (kdis35ckib + kdis35cki*Abeta)*p35CdkCKIn - (kexpckib +
kexpcki*Abeta)*CKIn - kasp35cki*p35Cdk5n*CKIn

# Erki represents the concentration of inactive Erk
Erki' = kierk*p35Cdk5c*Erka/(Jierk+Erka) - kaerk*Erki/(Jaerk+Erki)

# CycDT represents the total concentration of Cyclin D
```

```
CycDT' = kscycdb + kscycd*Erka - kdcycd*CycDT

# CycDCdk5c represents the concentration of cytoplasmic CycD-Cdk5 complex
CycDCdk5c' = kascycd*CycD*Cdk5c + kdiscyccki*CycCdkCKIc - (kdiscycd +
kascycdcki*CKIc + kdcycd)*CycDCdk5c

# CycDCdkCKIc represents the concentration of cytoplasmic CycD-Cdk5-CDKI complex
CycCdkCKIc' = kascycdcki*CKIc*CycDCdk5c - (kdiscyccki+ kdcycd)*CycCdkCKIc


# Set of algebraic expression

# p35c represents the concentration of free cytoplasmic p35
p35c = p35T - p35n - p35Cdk5n - p35CdkCKIn - p35Cdk5c - p35CdkCKIc
aux p35c = p35T - p35n - p35Cdk5n - p35CdkCKIn - p35Cdk5c - p35CdkCKIc

# Cdk5c represents the concentration of free cytoplasmic Cdk5
Cdk5c = Cdk5T - Cdk5n - p35Cdk5n - p35CdkCKIn - p35Cdk5c - p35CdkCKIc -
CycDCdk5c - CycCdkCKIc
aux Cdk5c = Cdk5T - Cdk5n - p35Cdk5n - p35CdkCKIn - p35Cdk5c - p35CdkCKIc -
CycDCdk5c - CycCdkCKIc

# CKIc represents the concentration of free cytoplasmic CDKI
CKIc = CKIT - CKIn - p35CdkCKIn - p35CdkCKIc - CycCdkCKIc
aux CKIc = CKIT - CKIn - p35CdkCKIn - p35CdkCKIc - CycCdkCKIc

Erka = ErkT - Erki
aux Erka = ErkT - Erki

# CycD represents the concentration of free cytoplasmic CyclinD
CycD = CycDT - CycDCdk5c - CycCdkCKIc
aux CycD = CycDT - CycDCdk5c - CycCdkCKIc

# p35nT represents the concentration of total nuclear p35 in either free, Cdk5 dimer or Cdk5-
CDKI trimer form
p35nT = p35n + p35Cdk5n + p35CdkCKIn
aux p35nT = p35n + p35Cdk5n + p35CdkCKIn

# p35cT represents the concentration of total cytoplasmic p35 in either free, Cdk5 dimer or
Cdk5-CDKI trimer form
p35cT = p35c + p35Cdk5c + p35CdkCKIc
aux p35cT = p35c + p35Cdk5c + p35CdkCKIc

# CKInT represents the concentration of total nuclear CDKI in either free or p35-Cdk5 trimer
form
CKInT = CKIn + p35CdkCKIn
aux CKInT = CKIn + p35CdkCKIn

@ total=2500,dt=0.5,method=stiff,bound=10000000
@ xlo=0,xhi=2500,ylo=0,yhi=1.6
```

```
@ NPLOT=4,yp1=Erka,yp2=CycCdkCKIc,yp3=p35CdkCKIn,yp4=p35Cdk5c
@ NTST=150,NMAX=20000000,NPR=5000,DS=-0.001
@ DSMAX=0.01,DSMIN=0.0001,PARMIN=0,PARMAX=1
@ AUTOXMIN=0,AUTOXMAX=0.02,AUTOYMIN=0,AUTOYMAX=1
done
```

XPPAUT code for cell cycle re-entry module 2 (Intracellular Ca^{2+}-dependent APC/C-Cdh1 inactivation, Rb hyperphosphorylation and E2F induction in neurons), related to STAR methods.

```
# Initial conditions for dynamic variables
init C=0.072,Cal=0.031,p25=0.116,Cdh1dp=0.987,Gls=0.063,CycB=0.01
init Glu=0.073,ROS=0.006,NADPH=0.567,GSH=1.916,E2FT=0.108
init Comp1=0.107,Rbpp=0.052


# Values of kinetic parameter
par Abeta=0.1,Cdh1T=1,GSHT=2, NADPT=1, p35T=2
par kinb=0.005, kinab=1, kinglu=0.4
par kinros=0.3, kout=0.5
par kscalb=0.001, kscal=0.4, ncc=2, khcal=0.5, kdcal=0.3
par ksp25=0.1, kdp25=0.05
par kicdh1=0.1, Jicdh1=0.1, kacdh1=0.1, Jacdh1=0.1
par ksgls=0.01, kdgls=0.01, kdglsc=0.15
par kscycb=0.001, kscyc=0.01, khcyc=0.25, kdcycb=0.01, kdcycbc=0.1
par ksglub=0.0001, ksglu=0.01, kdglu=0.01, Gln=1
par ksrosb=0.001, ksrosca=0.15, ksrosc=0.1
par kdros=0.15, kigsh=0.5,kagsh=0.5
par kanadphb=0.001, kanadphcdh=0.04, kinadph=0.01
par kse2fb=0.0003,kse2f=0.004,kde2f=0.003
par khe2f=0.25,RbT=1
par kase2f=100,kdise2f=1
par kpprb=0.25,kdprbpp=0.1
par Jpprb=0.05,Jdprb=0.05


# Set of differential equations:
# C represents the concentration of intracellular Ca2+ ion
C' = kinb + kinab * Abeta + kinglu * Glu + kinros * ROS- kout * C


# Cal represents concentration of activated calpain
Cal' = kscalb + kscal*C^ncc/(khcal^ncc + C^ncc) - kdcal*Cal


# p25 represents concentration of p25-Cdk5 complex
p25' = ksp25 * Cal * (p35T - p25) - kdp25 * p25


# Cdh1dp represents concentration of APC/C-Cdh1 in dephosphorylated form
Cdh1dp' = kacdh1 * Cdh1p/(Jacdh1 + Cdh1p) - kicdh1 * (p25 + CycB) *Cdh1dp/ (Jicdh1 + Cdh1dp)


# Gls represents concentration of glutaminase enzyme
Gls' = ksgls - (kdgls + kdglsc * Cdh1dp) * Gls
```

```
# CycB represents concentration of CycB-Cdk1 complex
CycB' = kscycb + kscyc*E2F/(khcyc + E2F) - (kdcycb + kdcycbc * Cdh1dp) * CycB

# Glu represents concentration of glutamate
Glu' = ksglub + ksglu * Gls * Gln - kdglu * Glu

# ROS represents concentration of reactive oxygen species
ROS' = ksrosb + ksrosca * C + ksrosc * CycB - kdros * ROS - kigsh * ROS * GSH^2

# Reduced NADPH concentration
NADPH' = (kanadphb + kanadphcdh * Cdh1dp)*(NADPT - NADPH)  - (kinadph +
kagsh*GSSG)*NADPH

# GSH represents concentration of glutathione in reduced form
GSH' = 2*kagsh*GSSG*NADPH - 2*kigsh*ROS*GSH^2

# E2FT represents the total concentration of E2F
E2FT' = kse2fb + kse2f*E2F/(khe2f + E2F) - kde2f*E2FT

# Comp1 represents complex between free E2F and Rb
Comp1' = kase2f*E2F*Rb - kdise2f*Comp1 - kpprb*(p25 + CycB)*Comp1/(Jpprb +
Comp1) - kde2f*Comp1

# Rbpp represents hyper-phosphorylated Rb (inactive form)
Rbpp' = kpprb*(p25 + CycB)*Rb/(Jpprb + Rb) + kpprb*(p25 + CycB)*Comp1/(Jpprb +
comp1) - kdprbpp*Rbpp/(Jdprb + Rbpp)

# Set of algebraic expression:
# Cdh1p is free form of phosphorylated Cdh1
Cdh1p = Cdh1T - Cdh1dp
aux Cdh1p = Cdh1T - Cdh1dp

# GSSG is the oxidized disulfide form of glutathione
GSSG = (GSHT - GSH)/2
aux GSSG = (GSHT - GSH)/2

# E2F is free form of the E2F
E2F = E2FT - Comp1
aux E2F = E2FT - Comp1
```

```
# Rb is active, dephosphorylated form of Rb
Rb = RbT - Comp1 - Rbpp
aux Rb = RbT - Comp1 - Rbpp


@ total=1500,dt=0.5,method=stiff,bound=10000000
@ xlo=0,xhi=1500,ylo=0,yhi=2.5
@ NPLOT=4,yp1=Cdh1dp,yp2=Rbpp,yp3=C,yp4=ROS
@ NTST=150,NMAX=20000000,NPR=5000,DS=-0.001
@ DSMAX=0.01,DSMIN=0.0001,PARMIN=-1,PARMAX=1
@ AUTOXMIN=0,AUTOXMAX=0.1,AUTOYMIN=0,AUTOYMAX=2.5
done
```

XPPAUT code for cell cycle re-entry module 3 (DNA damage dependent increase in CycD-Cdk4/6 and E2F accumulation in neurons), related to STAR methods.

```
# Initial conditions for dynamic variables
init p53T=0.374,p53aT=0,p53killer=0,Mdm2T=1.323
init ArfT=0.103,ArfMdm=0.087,p53DINP=0.06
init CycDT=0,CycET=0.216,E2FT=0.079,Comp1=0.078
init Rbpp=0.017,Ckit=0.2,CycECKI=0.183,CycDCKI=0,Ubl=0.02


# Values of kinetic parameter
par dnadamage=2.5,ksp53=0.5, kdp53p=0.1
par kap53=0.5, kip53=0.05
par kkip53=0.07, kkidp53=0.14, Jpp53=0.5, Jdpp53=0.1
par ksmdm2p=0.02, ksmdm2pp=0.3, kdmdm2p=0.1, kdmdm2pp=1
par ksarfp=0.01, ksarfpp=0.3, kdarf=0.1
par kasam=10, kdsam=2
par ksdinp1=0.0006, ksdinp2=0.016, ksdinp3=0.1, kddinp=0.01
par khdinp=0.5, khdinp1=0.5, khdinp2=1.2
par S=0, kscycds=0.008, kscycdg=0.01,kdcycd=0.008
par kscyceb=0.0002,kscyce=0.004,kdcyce=0.001
par khcyce=0.25
par kse2fb=0.0003,kse2f=0.004,kde2f=0.004
par khe2f=0.25,RBt=1
par kase2f=100,kdise2f=1
par kprb=5,kprbp=2,kpprbcyce=0.5,kpprb=0.25,kdprbp=1,kdprbpp=0.1
par p25=0
par Jprb=0.01,Jprbp=0.05,Jpprb=0.05,Jdprb=0.05
par ksckib=0.04,ksckip53h=0.2,ksckip53k=0,kdcki=0.2,kdckic=1
par kascki=100,kdiscki=0.1
par fac1=0.1,fac2=0.3
par ksubl=0.004,kdubl=0.002,kdublc=0.2
par Cdh1dp=1


# Set of differential equations:
# p53T represents the total concentration of p53
p53T'=ksp53-(kdp53p+kdp53pp*Mdm2)*p53T


# p53aT represents the total concentration of activated p53
p53aT'= kap53*dnadamage*(p53T - p53aT) - kip53*p53aT -
(kdp53p+kdp53pp*Mdm2)*p53aT


# p53killer represents the total concentration of p53 killer
p53killer' = kkip53*p53DINP*p53helper/(Jpp53+p53helper) -
kkidp53*p53killer/(Jdpp53+p53killer)-(kdp53p+kdp53pp*Mdm2)*p53killer
```

```
# Mdm2T represents the total concentration of Mdm2
Mdm2T'=ksmdm2p+ksmdm2pp*p53T-kdmdm2*Mdm2T


# ArfT represents the total concentration of Arf
ArfT'=ksarfp+ksarfpp*E2F-kdarf*ArfT


# ArfMdm represents the total concentration of Arf-Mdm2 complex
ArfMdm' = kasam*Arf*Mdm2 - kdsam*ArfMdm - kdmdm2*ArfMdm - kdarf*ArfMdm


# p53DINP represents the total concentration of p53DINP1
p53DINP' = ksdinp1 + ksdinp2*p53helper^3/(khdinp^3 + p53helper^3) +
ksdinp3*p53killer^3/(khdinp1^3 + p53killer^3)*E2F^3/(khdinp2^3 + E2F^3) -
kddinp*p53DINP

# CycDT represents the total concentration of Cyclin D
CycDT' = kscycds*S + kscycdg*dnadamage - kdcycd*CycDT


# CycET represents the total concentration of Cyclin E
CycET' = kscyceb + kscyce*E2F/(khcyce + E2F) - kdcyce*CycET


# E2FT represents the total concentration of E2F
E2FT' = kse2fb + kse2f*E2F/(khe2f + E2F) - kde2f*kde2fdnada*E2FT


# Comp1 represents complex between free E2F and Rb
Comp1' = kase2f*E2F*Rb + kdprbp*Comp2/(Jdprb + Comp2) - kdise2f*Comp1 -
kprb*CycD*Comp1/(Jprb + Comp1) - kpprbcyce*CycE*Comp1/(JppRb + Comp1) -
kpprb*p25*Comp1/(Jpprb + Comp1) - kde2f*kde2fdnada*Comp1


# comp2 represents complex between free E2F and Rb in mono-phosphorylated form
Comp2' = kase2f*E2F*Rbp + kprb*CycD*Comp1/(Jprb + Comp1) - kdise2f*Comp2 -
kdprbp*Comp2/(Jdprb+Comp2) - kprbp*(CycE + p25)*Comp2/(Jprbp + Comp2) -
kde2f*kde2fdnada*Comp2


# Rbp represents mono-phosphorylated Rb (active form)
Rbp' = kprb*CycD*Rb/(Jprb + Rb) + kdise2f*Comp2 + kde2f*kde2fdnada*Comp2 -
kase2f*E2F*Rbp - kdprbp*Rbp/(Jdprb + Rbp) - kprbp*(CycE + p25)*Rbp/(Jprbp + Rbp)


# Rbpp represents hyper-phosphorylated Rb (inactive form)
Rbpp' = kprbp*(CycE + p25)*Rbp/(Jprbp + Rbp) + kprbp*(CycE + p25)*Comp2/(Jprbp +
Comp2) + kpprbcyce*CycE*Rb/(Jpprb + Rb) + kpprbcyce*CycE*Comp1/(Jpprb + Comp1)
+ kpprb*p25*Rb/(Jpprb + Rb) + kpprb*p25*Comp1/(Jpprb + Comp1) -
kdprbpp*Rbpp/(Jdprb + Rbpp)
```

```
# Ckit represents the total concentration of CDKI
CKIT' = ksckib + ksckip53h*p53helper + ksckip53k*p53killer - (kdcki +
kdckic*CycE*Ubl)*CKIT

# CycECKI represents CDKI in complex with Cyclin E
CycECKI' = kascki*CycE*(CKIT-CycECKI-CycDCKI) - (kdiscki + kdcyce + kdcki +
kdckic*CycE*Ubl)*CycECKI

# CycDCKI represents CDKI in complex with Cyclin D
CycDCKI' = kascki*CycD*(CKIT-CycECKI-CycDCKI) - (kdiscki + kdcycd + kdcki +
kdckic*CycE*Ubl)*CycDCKI

# Ubl represents ubiquitin ligase
Ubl' = ksubl - (kdubl + kdublc*Cdh1dp)*Ubl

# Set of algebraic expression:
# E2F is free form of the E2F
E2F = E2FT - Comp1 - Comp2
aux E2F = E2FT - Comp1 - Comp2

# Rb is active, dephosphorylated form of Rb
Rb = RbT - Comp1 - Comp2 - Rbp - Rbpp
aux Rb = RbT - Comp1 - Comp2 - Rbp - Rbpp

# CycE is free form of Cyclin E
CycE = CycET - CycECKI
aux CycE = CycET - CycECKI

# CycD is free form of Cyclin D
CycD = CycDT - CycDCKI
aux CycD = CycDT - CycDCKI

# Arf is free form of Arf
Arf=ArfT-ArfMdm
aux Arf=ArfT-ArfMdm

# Mdm2 is free form of Mdm2
Mdm2=Mdm2T-ArfMdm
aux Mdm2=Mdm2T-ArfMdm
```

```
# p53helper is activated p53 in helper form
p53helper=p53aT-p53killer
aux p53helper=p53aT-p53killer


# Effect of DNA damage on p53 stability represented by change in degradation rate
kdp53pp=1/(1+fac1*dnadamage)
# Effect of DNA damage on Mdm2 stability represented by change in degradation rate
kdmdm2=kdmdm2p+kdmdm2pp*dnadamage

# Effect of DNA damage on E2F stability represented by change in degradation rate
kde2fdnada=1/(1+fac2*dnadamage)


@ total=3500,dt=0.5,method=stiff,bound=10000000
@ xlo=0,xhi=3500,ylo=0,yhi=4
@ NPLOT=5,yp1=Rbpp,yp2=E2F,yp3= p53killer,yp4=CycDT,yp5=Ckit
@ NTST=150,NMAX=20000000,NPR=5000,DS=-0.002
@ DSMAX=0.01,DSMIN=0.0001,PARMIN=0,PARMAX=2.5
@ AUTOXMIN=0,AUTOXMAX=2.5,AUTOYMIN=0,AUTOYMAX=2.5


done
```
